# The impact of hypoxia and oxidative stress on proteo-metabolomic alterations of 3D cholangiocarcinoma models

**DOI:** 10.1038/s41598-023-30204-y

**Published:** 2023-02-21

**Authors:** Pimpawadee Phukhum, Jutarop Phetcharaburanin, Kwuanjira Chaleekarn, Yingpinyapat Kittirat, Thanaporn Kulthawatsiri, Nisana Namwat, Watcharin Loilome, Narong Khuntikeo, Attapol Titapun, Arporn Wangwiwatsin, Tueanjit Khampitak, Manida Suksawat, Poramate Klanrit

**Affiliations:** 1 Department of Biochemistry, Faculty of Medicine, Khon Kaen University, Khon Kaen, 40002 Thailand; 2 Khon Kaen University Phenome Centre, Khon Kaen, 40002 Thailand; 3 Cholangiocarcinoma Research Institute, Khon Kaen University, Khon Kaen, 40002 Thailand; 4 Department of Surgery, Faculty of Medicine, Khon Kaen University, Khon Kaen, 40002 Thailand

**Keywords:** Metabolomics, Cancer metabolism, Cancer models

## Abstract

The three-dimensional multicellular spheroid (3D MCS) model has been employed in cholangiocarcinoma research as it generates 3D architecture and includes more physiological relevance with the multicellular arrangement. However, it is also essential to explain the molecular signature in this microenvironment and its structural complexity. The results indicated that poorly differentiated CCA cell lines were unable to form 3D MCS due to the lack of cell adhesion molecules with more mesenchymal marker expression. The well-differentiated CCA and cholangiocyte cell lines were able to develop 3D MCSs with round shapes, smooth perimeter, and cell adhesion molecules that led to the hypoxic and oxidative microenvironment detected. For MMNK-1, KKU-213C, and KKU-213A MCSs, the proteo-metabolomic analysis showed proteins and metabolic products altered compared to 2D cultures, including cell–cell adhesion molecules, energy metabolism-related enzymes and metabolites, and oxidative-related metabolites. Therefore, the 3D MCSs provide different physiological states with different phenotypic signatures compared to 2D cultures. Considering the 3D model mimics more physiological relevance, it might lead to an alternate biochemical pathway, targeting to improve drug sensitivity for CCA treatment.

## Introduction

Cholangiocarcinoma (CCA) is a malignant tumor of the bile duct epithelium. It has been highly endemic in the Northeast of Thailand. Due to the poor early diagnostic power, most patients present the symptoms at the advanced stage, and the five-year survival rate is considered relatively low^1^. Currently, cancer research primarily relies on in vitro (2D culture) model to understand cancer biology. However, three-dimensional (3D) multicellular spheroid (MCS) or 3D spheroid is believed to possess an additional potential for drug evaluation regarding the 3D multicellular structure^2^. 3D MCS is an in vitro cell culture that simulates a 3D micro-tissue and has more physiological relevance to disease-specific microenvironment^3^. It can be created into 3D cell aggregates from the single or multicell types using various scaffold-based or scaffold-free techniques^4^. 3D MCSs have several advantages for biomedical research; for example, 3D MCS grow in their 3D physiological shape and represent multicellular arrangement and extracellular matrix (ECM). In addition, 3D MCSs allow better cell-to-cell interactions, intercellular signaling pathways, gene expression, and more structural complexity^5^. Moreover, they provide different cellular microenvironments, including typical zonation of proliferative, quiescent, and necrotic areas, that have gradient distributions of diffused oxygen, nutrients, CO_2_, and metabolic waste in the structure^6^. Therefore, forming multilayers allow the MCSs to resist certain drugs due to their metabolic changes and the physical barrier in a 3D environment. For the metabolic changes, we could explore the cellular or molecular mechanism by using metabolomic analysis.

The metabolomic analysis is a powerful tool for profiling the biochemical composition and exploring the metabolic pathways, particularly within the cells^7^. The categories of metabolomics analyses include targeted or untargeted analysis. The untargeted analysis focuses on the metabolic profiling of the total complement of metabolites and “fingerprint” in a sample. In contrast, the targeted analysis focuses on the quantification and identification of selected metabolites. Although various analytical techniques have been used, nuclear magnetic resonance (NMR) spectroscopy and mass spectrometry (MS) are the widely used tools in metabolomics research^7^. In the current study, we employed the ultra-high-performance liquid chromatography-mass spectrometry (UHPLC-MS)-based metabolomics which has high sensitivity and specificity and is superior in allowing analysis of both primary and secondary metabolites^8^. The complete set of cellular metabolites between the 2D culture system and 3D MCS were distinguished. The alteration of both essential and non-essential amino acids was found to be greater in 3D MCS compared with those in 2D culture. Furthermore, there were higher activities of glycolysis and degradations of nucleic acids, proteins, and amino acids in 3D MCSs. The lower tricarboxylic acid cycle (TCA) flux and diminishing glutathione (GSH) and its precursors were observed.

The distinct metabolomic features in 2D cells and 3D MCSs may benefit metabo-phenotypic targeting for CCA treatment. Therefore, we hypothesized that the specific alteration of metabolites in 2D cells and 3D MCS from CCA and cholangiocyte cell lines might lead to certain biochemical pathway targeting to improve chemotherapy sensitivity. To confirm the relevance, the expression levels of the proteins and enzymes in the glycolytic pathway and TCA cycle, including glucose transporter-1 (GLUT-1), glyceraldehyde 3-phosphate dehydrogenase (GAPDH), lactate dehydrogenase A (LDHA), isocitrate dehydrogenase 1 (IDH1), isocitrate dehydrogenase 2 (IDH2) and vascular endothelial growth factor C (VEGF-C) were investigated. Combining all data together, the related proteo-metabolomic profile of each cell line and culture condition is revealed. The unique proteo-metabolomic signatures of 3D MCSs were physiologically relevant and may provide insight into novel drug- targeting for CCA treatment.

## Results

### Fabrication of 3D multicellular spheroid (MCS) using adapted agarose-coating method (U-surface)

**Figure 1:**
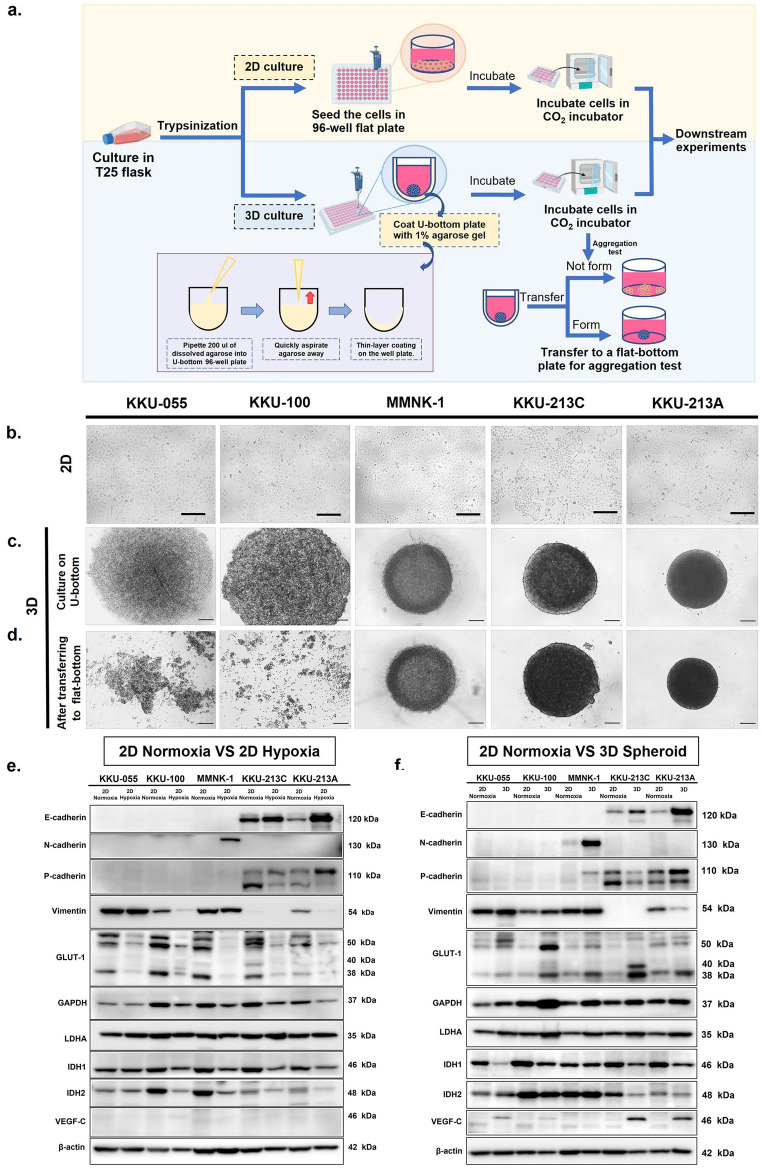
Formation of 3D MCSs using five CCA and cholangiocyte cell lines. (**a**) The process of agarose-coating (U-surface), showing the coating procedures. The illustration was created with BioRender.com. (**b**) Four CCA cell lines (KKU-055, KKU-100, KKU-213C, KKU-213A) and one cholangiocyte cell line (MMNK-1) are shown. Each cell line is in a 2D monolayer culture system. (**c**) 3D cultures of 5 cell lines in the coated U-bottom plate, incubated for 48 h. (**d**) Cells transferred back to the flat-bottom plate, showing only KKU-055 and KKU-100 scattered to single cells, while the rest maintain intact spherical shape as 3D MCS, scale bar represents 200 µm. (**e** and **f**) Western blot analysis of cell-cell adhesion molecules (E-cadherin, N-cadherin, and P-cadherin), mesenchymal marker (vimentin), enzymes involved in the glycolytic pathway (GLUT-1, GAPDH, and LDHA), enzymes related to TCA cycle (IDH1 and IDH2), and angiogenic factor (VEGF-C) express on 2D normoxia vs. 2D hypoxia (**e**) and 2D normoxia vs. 3D spheroid (**f**) of CCA and cholangiocyte cell lines. Quantitative analysis of protein (normalized to β-actin) is shown in supplement S1. Data are means ± S.D. of at least three independent experiments. *P < 0.05, **P < 0.01 and ****P < 0.01.

The agarose-coating scheme can prevent cell adhesion to the tissue-culture surface as demonstrated by several publications^9–12^. Therefore, the cells aggregate together as spherical-like structures^13^. Agarose is usually coated on the flat surface (e.g., 96-well plate, flat-bottom) to obtain a slight concaved surface, which helps inhibit cell adhesion while sedimentation and promote cell aggregation. Although agarose-based coating has been reported on flat-bottom^9^, low amount of agarose is placed into the well statically without aspiration (also be applied to the U-bottom plate). Our method (Fig. 1a) has adapted this scheme to create a dynamic agarose coating on the U-bottom well instead. 200 µL of agarose was placed in a U-bottom well and promptly aspirated to create a very thin gelated layer along with the U-surface, which improves the centering and aggregation of cells better than a static flatbed coating. Our results showed the cell could be seeded straight away after finish coating and the minimum cell seeding could be as low as 1000 cells per well with highly uniformity (Supplementary Fig. 1). Contrary to the poly-HEMA coating, which requires a longer coating time, this technique creates a ready-to-use plate for cell seeding straight away. In contrast, the poly-HEMA method requires up to 3 days to complete ethanol evaporation for the coating step^14^.

### Some cholangiocarcinoma and cholangiocyte cell lines form 3D multicellular spheroid (MCS), and cell adhesion molecules affect intact spheroids

Four cholangiocarcinoma (KKU-055, KKU-100, KKU-213C, and KKU-213A) cell lines and one cholangiocyte cell line (MMNK-1) were chosen for 3D MCS formation in vitro compared to the 2D monolayer culture (Fig. 1b). The confirmation of 3D cell aggregation was performed by transferring the cells in U-bottom plates back to flat-bottom plates and observing the immediate disaggregation to single cells when MCSs did not form. The results showed that MMNK-1, KKU-213C, and KKU-213A remained spherical even when transferring back to the flat-bottom plate (Fig. 1c and d). These results may relate to their characteristics of moderately to well-differentiated epithelial phenotypes that still express cell adhesion molecules (Supplementary Table 1). However, KKU-055 and KKU-100 aggregated at the center of the U-bottom plate but separated into individual cells promptly after transferring back to the flat-bottom plate (Fig. 1c, d). This may be due to their poorly differentiated phenotypes, confirmed by high vimentin expression (Fig. 1e). Later, we investigated the cell adhesion molecules via western blotting, including E-cadherin, N-cadherin, and P-cadherin (Fig. 1e). These molecules may play roles in the cell adhesion of 3D MCSs. The results showed that KKU-055 and KKU-100 did not express all 3 cadherins in 2D and 3D cultures that were relevant to the previous publication (2D KKU-100)^15^. The lacks of cell adhesion molecules were relevant to the inability of MCS formation of these cell lines. For MMNK-1, the molecules that enhanced cell aggregation were N-cadherin and P-cadherin, of which their expressions were higher in 3D MCS. In contrast, KKU-213C and KKU-213A showed E-cadherin and P-cadherin expressions in 2D and 3D cultures, while N-cadherin did not seem to play a role in these two cell lines. For E-cadherin, higher expression levels were observed in both 3D KKU-213C and 3D KKU-213A. These results conformed with their capabilities of MCS formation as E-cadherin is necessary for spheroid integrity^16^. In contrast, P-cadherin had higher expression levels in the 3D culture of KKU-213A but low expression in 3D KKU-213C. Therefore, each cell line may have different biochemical phenotypes determined from co-expression or no expression of varying adhesion molecules. Those variations were also magnified or reduced when forming 3D MCSs.

### The 3D MCSs of CCA and cholangiocyte cell lines alter their protein expressions when the microenvironment changed

Cholangiocarcinoma (KKU-055, KKU-100, KKU-213C, and KKU-213A) cell lines and cholangiocyte cell line (MMNK-1) were employed for the investigation of protein expressions that differentiate 3D MCS from a 2D culture system. Although KKU-055 and KKU-100 did not form 3D structures, they could experience protein alterations via the cell compactness caused by the U-bottom plate (3D culture). The level of a transmembrane protein and glycolytic enzymes, including glucose transporter 1 (GLUT-1), glyceraldehyde 3-phosphate dehydrogenase (GAPDH), and lactate dehydrogenase A (LDHA), were investigated through western blotting. The results show that GLUT-1 was expressed via 40–60 kDa^17^ with both non-glycosylated (38–40 kDa) and glycosylated forms (50–60 kDa)^17^, with upregulations in 3D cultures (Fig. 1f). GAPDH and LDHA expressions involved in glycolytic enzyme activity also increased in the 3D cultures of all cell types (Fig. 1f). In addition, vascular endothelial growth factor C (VEGF-C) related to angiogenic activation^18^ was upregulated in the 3D cultures of CCA cell lines (Fig. 1f). In contrast, the enzymes involved in the cycle, namely isocitrate dehydrogenase 1 and 2 (IDH1 and IDH2), were downregulated in some 3D CCA cells but not in MMNK-1 (Fig. 1f).

The hypoxia-induced 2D cultures (1% O_2_) were employed for hypoxic controls (Fig. 1e). Notably, the alterations of protein expression were also observed under this condition with similar or different patterns to 3D MCS. There were no or very low expressions of cell adhesion molecules in KKU-055 and KKU-100. For MMNK-1, E- and P-cadherins were not observed in all conditions; however, N-cadherin was expressed more when cultured in 2D hypoxia (similar to 3D MCS). For KKU-213C and KKU-213A, E-cadherin was increased in 2D hypoxia, while N-cadherin did not have any expression in both cell lines. Moreover, P-cadherin has lower expression in KKU-213C, while having more expression in KKU-213A. For vimentin, overall trends of each cell line were similar between 2D hypoxia and 3D culture except for KKU-100 which vimentin was downregulated in 2D hypoxia while it was upregulated in 3D culture.

For GLUT-1 expression, the trend seemed different compared 2D hypoxia to 3D MCS. While glycolytic enzymes (GLUT-1, GAPDH, and LDHA) increased expressions in 3D MCSs, the hypoxic 2D cells reduced GLUT-1 and GAPDH expressions but exhibited no difference in LDHA. For IDH1 and IDH2, they were consensually decreased in all hypoxic 2D cells similar to 3D cells except for 3D MMNK-1 which had no difference. For VEGF-C, the expression increased in 3D cultures of CCA cell lines (KKU-055, KKU-100, KKU-213C, and KKU-213A) but not MMNK-1 while there was no obvious expression in normoxic and hypoxic 2D cells (Fig. 1e).

These results may indicate that 3D culture systems induce the alterations to undergo more glycolytic and angiogenic activities while reducing the TCA cycle activity, resulting in higher production and accumulation of lactate which is the cancer signature^19^. The relevant findings were confirmed in our metabolomic study.

### The agarose-coating method (U-surface) is robust and reveals a uniform size of MCS’s projected area, which correlates with initial seeding cell numbers

**Figure 2:**
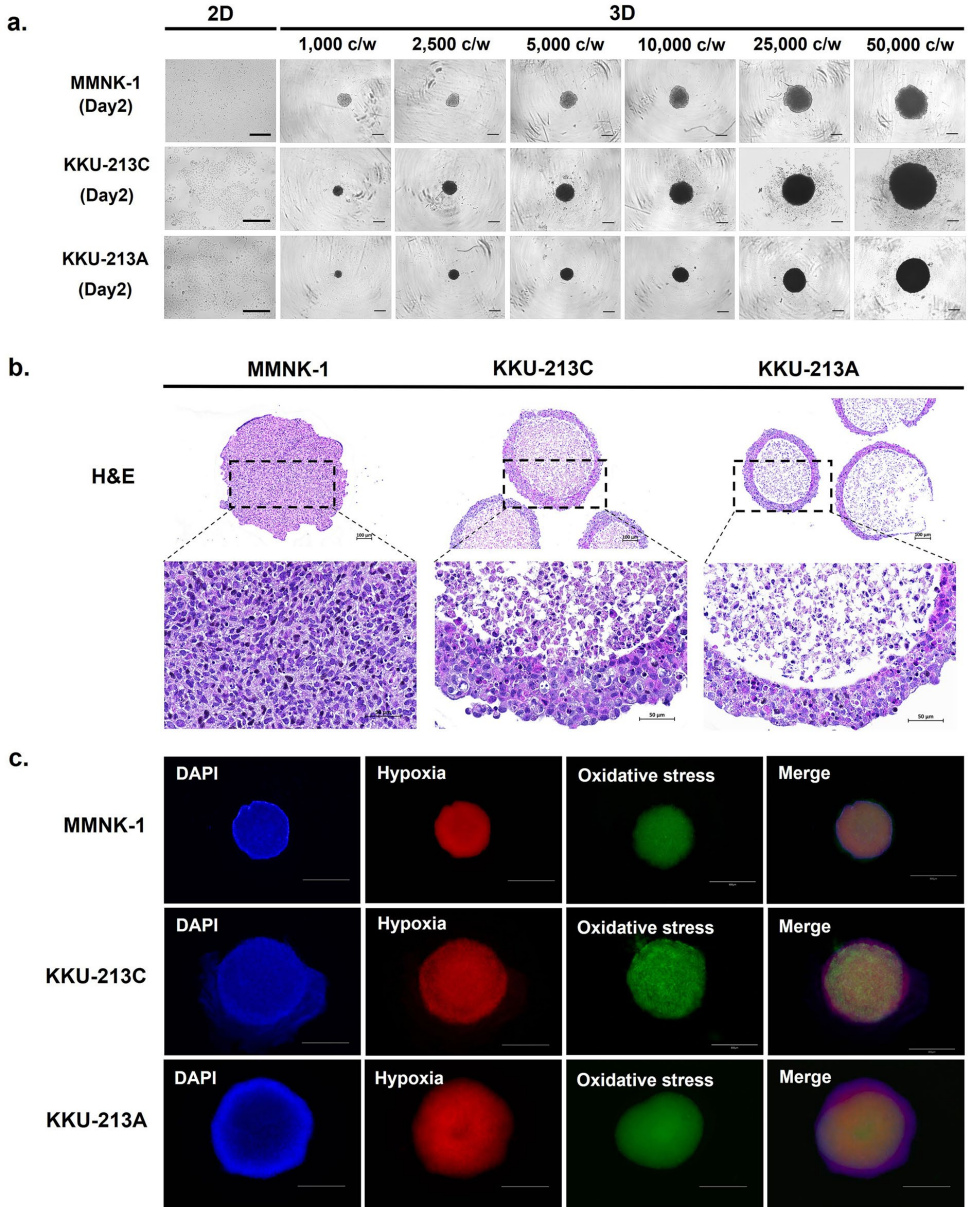
Characterization of 3D MCSs. (**a**) CCA and cholangiocyte cell lines were varied with seeding numbers (1,000 2,500 5,000 10,000 25,000 and 50,000 cells per well) and cultured for 48 h to observe the MCS aggregation under an inverted microscope. Scale bar represents 500 µm. (**b**) The 3D MCSs were embedded, sectioned, and stained with H&E. Scale bar represents 100 µm and 50 µm for zoom-in pictures. (**c**) The 3D MCSs exhibit typical zonation of hypoxia and oxidative stress determined by nuclei (DAPI, blue), Hypoxia probe (red), Oxidative stress probe (green). Scale bar represents 800 µm.

Two CCA cell lines and one cholangiocyte cell line (KKU-213C, KKU-213A, and MMNK-1, respectively) were selected for further investigation to evaluate the reproducibility of MCSs. Varying cell numbers (1000 2500 5000 10,000 25,000 and 50,000 cells/well) were seeded and cultured for 48 h to observe the MCSs aggregation under an inverted microscope (Fig. 2a). The images were analyzed using the ImageJ program to measure the projected area, circularity, and roundness to describe the quality of MCS. The results showed the projected area was positively correlated with cell number with uniformity with the R^2^ = 0.9852, 0.9425, and 0.9926 for MMNK-1, KKU-213C, and KKU-213A, respectively (Supplementary Fig. S2a). MCSs of all cell concentrations had both circularity and roundness above 0.8. This indicated that all MCSs had round shapes with a smooth perimeter rather than an irregular shape (Supplementary Fig. S2b and S2c). The spheroid can be cultured until day 7, although the dramatic change in sizes was observed when the cell number is higher than 5000 cells per spheroid (Supplementary Fig. S1).

### Cholangiocarcinoma and cholangiocyte MCSs exhibit typical zonation of hypoxia and oxidative stress

3D MCSs of CCA and cholangiocyte were characterized by morphology. Paraffin-embedded samples were processed with H&E staining (Fig. 2b). MMNK-1 showed the cross-sections that were irregular shapes. This may be the result of the loose aggregation of cells with processing through the dehydration process but reveal the distribution of cells across the spheroid mass. However, KKU-213C and KKU-213A indicated a nearly circular section with a compact outermost but less dense inner layer. Therefore, we further examined using hypoxia and oxidative stress probes that detect hypoxia-induced nitroreductase activity (red) and reactive nitrogen and oxygen species (green). All MMNK-1, KKU-213C, and KKU-213A expressed the positive signals of hypoxia and oxidative stress probes at the inner layer of MCSs (Fig. 2c). The results revealed that all MCSs were affected by hypoxia and oxidative stress at the inner core due to 3D structure formation. The indication of a thin unaffected zone with only blue nuclei was observed at the outmost layer of all MCSs. In contrast, the hypoxia and oxidative stress signals were relatively low in normoxia 2D cultures, which may be due to the good exposure to oxygen in a monolayer manner. Whereas the positive indications of both probes were examined in hypoxia 2D (Supplementary Fig. S4). The global metabolomic analysis was further investigated to elaborate on the active metabolites involved in the process.

### Global metabolomic profiles are distinctive between 2D culture and 3D MCS and among different cell lines

**Figure 3:**
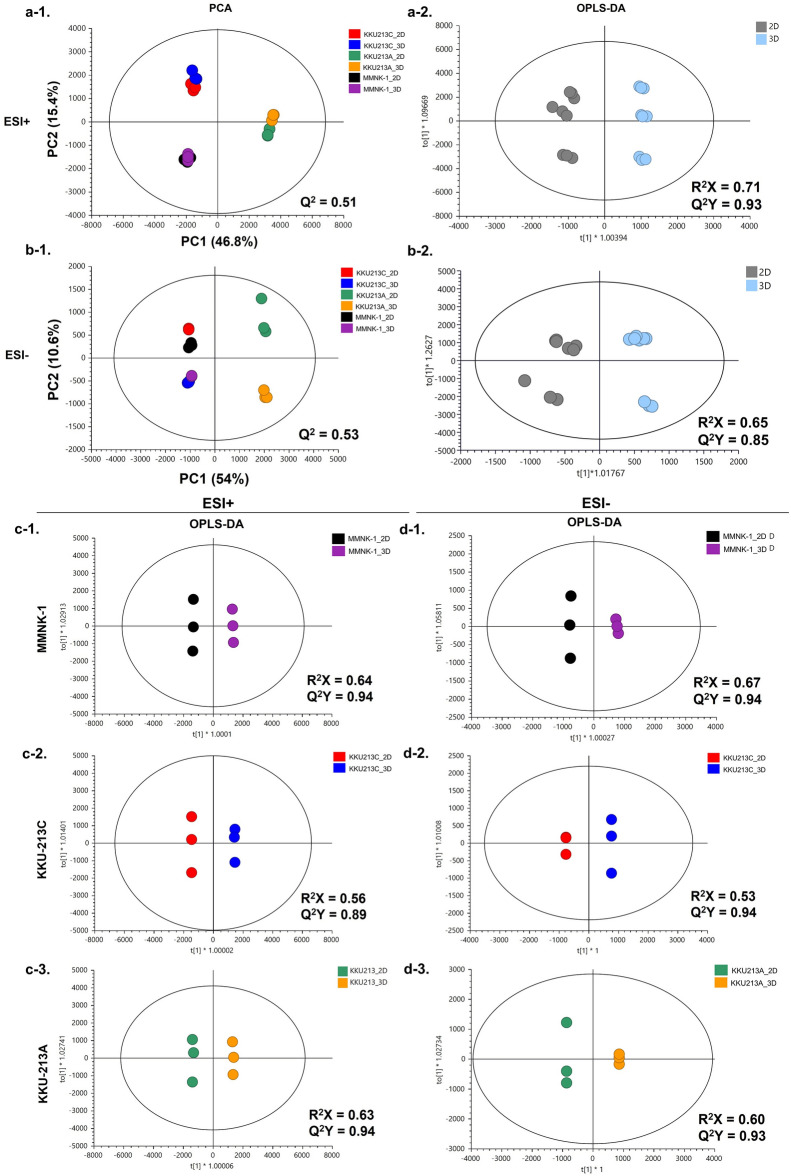
Multivariate statistical analysis based on the metabolomic profiling data sets of 2D culture and 3D MCS of CCA and cholangiocyte cell lines. (**a-1**, **b-1**) PCA score plots and (**a-2**, **b-2**) O-PLS-DA score plots based on UHPLC-ESI-Q-TOF-MS/MS data set of 2D and 3D from CCA and cholangiocyte cell lines**.** For individual cell line analysis, the left and right panels represent ESI + mode (1) and ESI- mode (2). (**c**) O-PLS-DA score plots base on UHPLC-ESI-Q-TOF-MS/MS data sets of 2D vs. 3D detected in ESI + ionization mode. (**d**) O-PLS-DA score plots base on UHPLC-ESI-Q-TOF-MS/MS data sets of 2D vs. 3D detected in ESI- ionization mode. MMNK-1 presented in c-1 and d-1, KKU-213C presented in c-2 and d2, and KKU-213A presented in c-3 and d-3. The figures were plotted using SIMCA-14.1 (Umetrics, Sartorius Stedim Biotech).

UHPLC-MS-based metabolomic profiling was conducted on cellular extracts in both positive and negative electrospray ionization (ESI + and ESI−, respectively) modes to gain insight into the molecular mechanisms that govern the metabolic fates between different cells and culture systems. In ESI + mode, the principal component analysis (PCA) scores plot reveals that different cell lines exhibited distinct metabolic characteristics (Fig. 3a-1). Both 2D and 3D of KKU-213A cells were clearly separated from those of KKU-213C and MMNK-1 cells along the first principal component (PC1) and the second principal component (PC2) determined clear class separation between KKU-213C and MMNK-1 in both 2D cultures and 3D MCSs (Fig. 3a-1). Likewise, PCA scores plot of ESI- mode followed the similar trends with a greater variation between 2D cultures and 3D MCSs of KKU-213A cells (Fig. 3b-1). To visualize metabolic differences between 2D cultures and 3D MCSs, pairwise orthogonal partial-least square discriminant analysis (O-PLS-DA) models were constructed on spectral data obtained from both 2D and 3D systems of all cell lines. It is clearly seen that 2D cultures and 3D MCSs possessed different metabolic signatures regardless of different cell lines in both ESI + mode (Fig. 3a-2) and ESI- mode22 (Fig. 3b-2).

**Figure 4:**
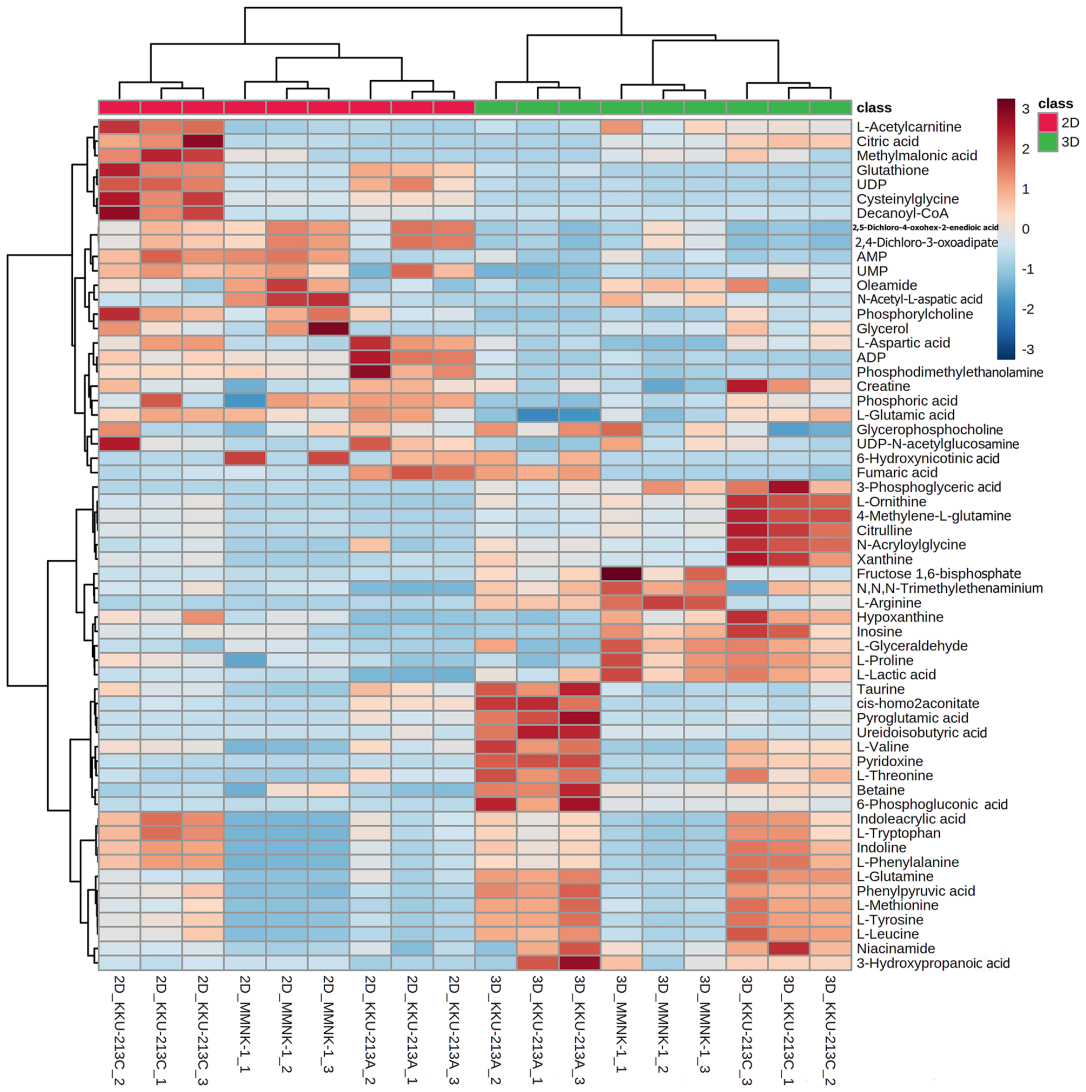
Heat map of differential metabolites of each cell line in 2D or 3D systems. Z-score hierarchical clustering based on Euclidean distance measure and Ward’s method for linkage analysis. Each row represents one of 59 metabolites, and each column represents one sample (culture system_cell line_experiment number). The color scale means the metabolite intensity standard deviations from the mean, with blue for low intensity and red for high-intensity levels. The figure was generated by MetaboAnalyst 5.0 (https://www.metaboanalyst.ca/)^21^ using our own data as input.

To elucidate the metabolomic differences between 2D culture and 3D MCS in each cell line, PCA and O-PLS-DA pairwise comparison models were constructed. PCA scores plots of 2D and 3D systems of all cell lines show the distinct separations along PC1 in both ESI + and ESI- modes (Supplementary Fig. S5). O-PLS-DA models between 2D cultures, and 3D MCSs of all cell lines in both ionization modes were valid (permutation *p* < 0.05, Supplementary Fig S16). The loading plots of all pairwise PCA models (Supplementary Fig. S17) were crossed-checked, and the discriminatory metabolites are very similar to those reported by O-PLS-DA S-plots. Consistently, univariate analysis revealed the conforming statistical significance of those metabolites (Supplementary Fig. S7–S12). The scores plots showed the prominent class separations (Fig. 3c and d). In addition, O-PLS-DA corresponding S-line plots (Supplementary Fig. S6) demonstrated significant discriminatory metabolites for all pairwise comparisons that are summarized in (Supplementary Table S3 and Supplementary Fig. S7–S12). A total of 59 altered metabolites were further analyzed using hierarchical clustering based on Euclidean distance measure and Ward’s method for linkage analysis^20^ (Fig. 4). The metabolic shifts from 2 to 3D culture systems were clearly seen through altering levels of metabolites independent of cell types (Fig. 4). 3D MCSs of KKU-213A and KKU-213C exhibited elevated levels of branched-chain amino acids (BCAAs) (*e.g.*, L-valine and L-leucine), aromatic amino acids (e.g., L-tryptophan, L-tyrosine, and L-phenylalanine), sulfur-containing amino acid (*e.g.*, methionine), amidic amino acid (*e.g.*, L-glutamine), hydroxylic amino acid (*e.g.*, L-threonine), indoles (*e.g.*, indoline and indoleacrylic acid), pentose phosphate pathway intermediate (*e.g.*, 6-phosphogluconic acid), phenylalanine pathway intermediate (*e.g.* phenylpyruvic acid), BCAA and propionic acid breakdown product (*e.g.*, 3-hydroxypropanoic acid) and vitamins (*e.g.*, pyridoxine and niacinamide) compared with their 2D cultures (Fig. 4).

It is also noteworthy that high relative concentrations of intracellular taurine, pyroglutamic acid, cis-homo-2-acotinic acid, pyroglutamic acid, and ureidoisobutyric acid were observed in the 3D MCS of KKU-213A only. The metabolic shifts from 2D culture to 3D MCS of KKU-213C were prominently observed through the decreased levels of tricarboxylic acid cycle (TCA) intermediates (*e.g.,* citric acid) and its acetyl-CoA donors (*e.g.,* L-acetylcarnitine, methylmalonic acid, and decanoyl CoA). 3D MCSs of MMNK-1 and KKU-213C showed higher relative concentrations of *N*,*N*,*N*-trimethylethenaminium, hypoxanthine, inosine, L-glyceraldehyde, L-proline, and L-lactic acid. While levels of fructose-1,6-bisphosphate and L-arginine were found higher only in 3D MMNK-1, 3D KKU-213C contained remarkably higher levels of 3-phosphoglyceric acid, 4-methylene-L-glutamine, L-ornithine, citrulline, and xanthine (Fig. 4).

It was noticeable that the antioxidant system, namely glutathione (GSH) and its precursor, were reduced probably due to the increased oxidative stress and led to evident necrotic cores of 3D KKU-213A and KKU-213C (Fig. 2b). The intermediates of purine and pyrimidine degradation were also detected with increased inosine, hypoxanthine, xanthine, and ureidoisobutyrate. These results were confirmed with RNA integrity analysis via Agilent 4200 Tapestation System (Supplementary Fig. 12). The RNA integrity numbers (RIN) were reduced in all 3D MCSs compared to 2D culture, as well as electropherograms showed a higher degradation pattern of total RNA in all MCS models.

**Figure 5:**
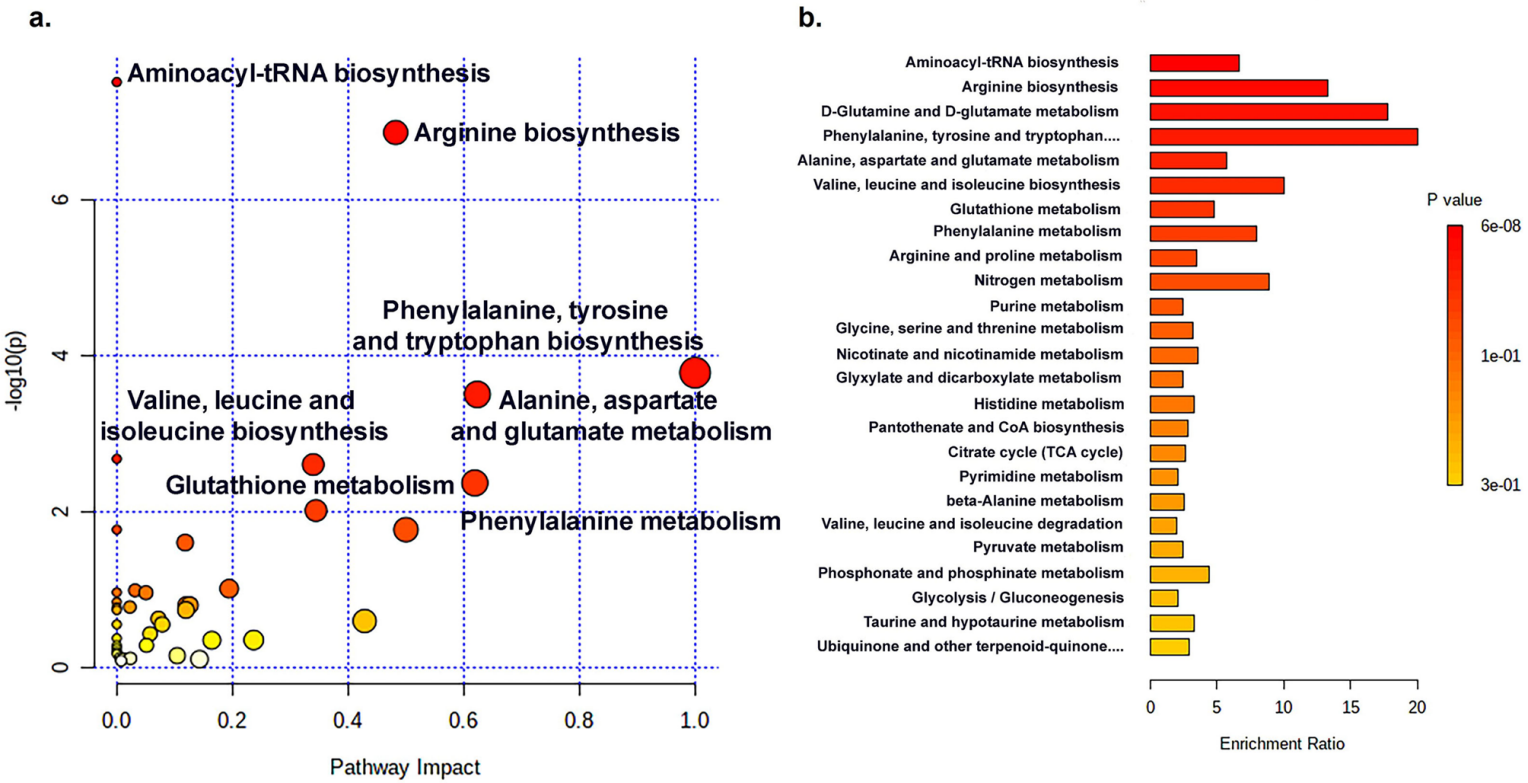
Summary of pathway analysis on 2D culture and 3D MCS, analyzed using MetaboAnalyst 5.0. To define the influence pathway, all metabolites in 2D culture and 3D MCS on ESI + and ESI- were elucidated by pathway analysis. (**a**). Metabolic pathway analysis on 2D culture and 3D MCS. (**b**). Enrichment analysis on identified metabolite in 2D culture and 3D MCS from CCA and cholangiocyte cell lines. The figure was generated by MetaboAnalyst 5.0 (https://www.metaboanalyst.ca/)^21^ using our experimental data as input.

**Figure 6:**
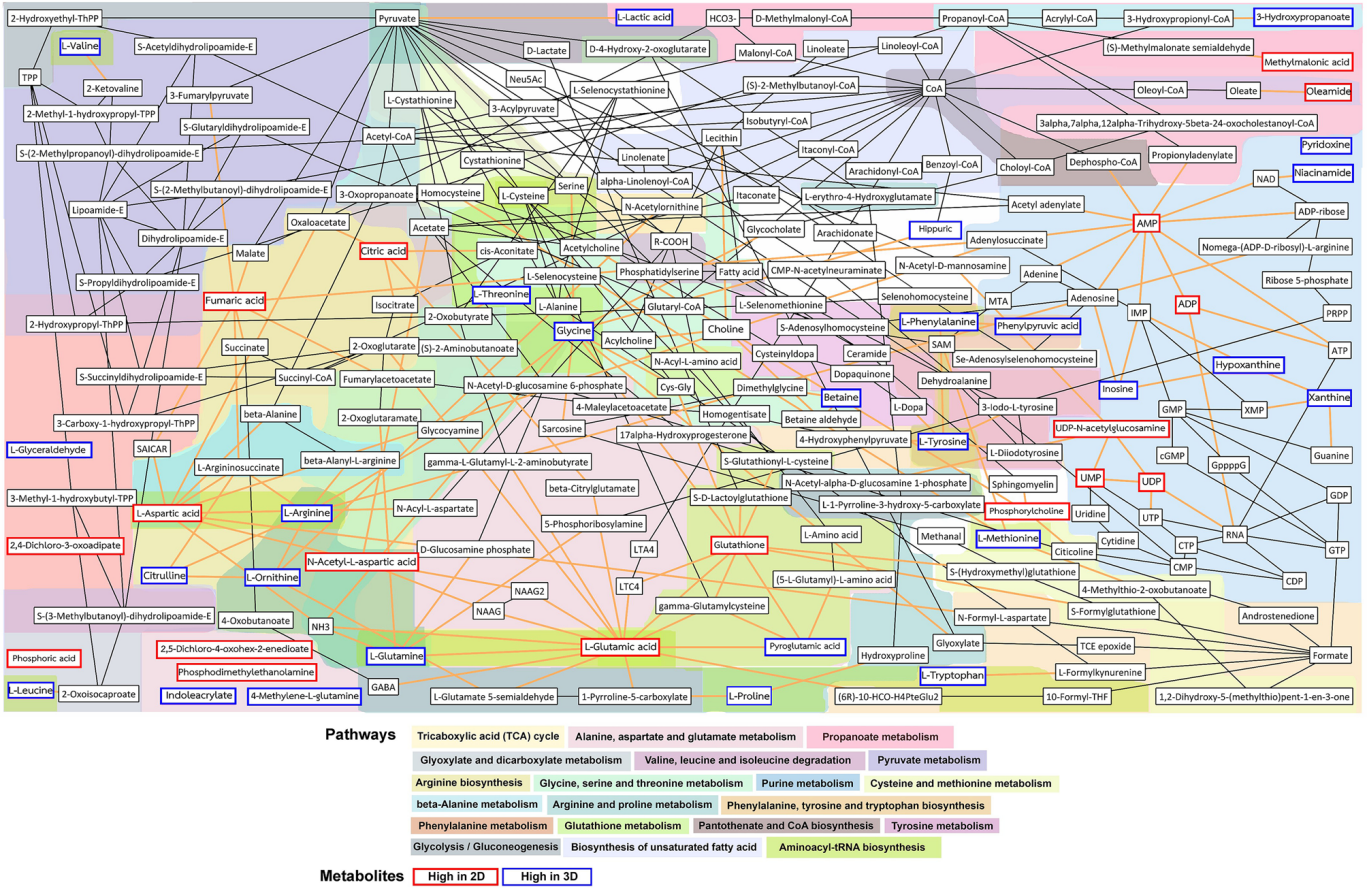
The impact of 2D and 3D culture systems on CCA and cholangiocyte cell lines metabolic reaction networks. Metabolic reaction networks of metabolites found in different 2D and 3D in CCA and cholangiocyte cell lines comparisons, using KEGG pathway-based^24–26^ MetaboNetworks toolbox in MATLAB environment. The networks show metabolic reaction links between metabolites in each cell line. The red-line boxes indicate the metabolites found with higher concentrations in the 2D culture, while the blue-line boxes indicate those with higher concentrations in 3D MCS. The black-line boxes indicate metabolites in the metabolic backbone. The colored-background shading indicates different metabolic pathways. This metabolic map was generated using MATLAB R2015a (MathWorks, Inc., USA) equipped with MetaboNetwork toolbox^23^ using our own data as input and the background shading was added to the graphs to indicate the different interconnecting pathways^27^.

To identify the metabolic pathways perturbed by different culture systems, all 59 attributed metabolites were analyzed with a web-based metabolomics tool for pathway analysis and visualization (MetPA)^22^. The most influenced pathways are shown in Fig. 5. The most influenced pathway was ascribed a pathway impact of > 0.1 and − log(*p*) > 2. Four metabolic pathways were found: aminoacyl-tRNA biosynthesis; arginine biosynthesis; phenylalanine, tyrosine, tryptophan biosynthesis; alanine, aspartate, and glutamate metabolism. The detailed metabolic pathways are listed in Table 1. These results prompted the reprogramming of metabolic networks influenced by different culture systems. As perturbations to a living system often instigate changes to multiple pathways simultaneously, we show here a condensed multi-compartmental metabolic reaction network of homeostatic cellular signature of differences between 2D and 3D culture systems^23^ (Fig. 6).

**Table 1:** The summary of pathway impact on 2D culture and 3D MCS.

Pathways	Hits	Raw *p*	Holm adjust	FDR	Impact
Aminoacyl-tRNA biosynthesis	12	3.08E-08	2.58E-06	2.58E-06	0
Arginine biosynthesis	7	1.36E-07	1.13E-05	5.73E-06	0.48223
Phenylalanine, tyrosine, and tryptophan biosynthesis	3	0.00017	0.01354	0.00462	
Alanine, aspartate, and glutamate metabolism	6	0.00031	0.0253	0.00655	0.6234
Valine, leucine, and isoleucine biosynthesis	3	0.00208	0.1671	0.03452	0
Glutathione metabolism	5	0.00246	0.1948	0.03452	0.33951
Phenylalanine metabolism	3	0.00425	0.3317	0.05104	0.61904

**Hits** is the matched number from the user uploaded data; **Raw p** is the original *p* value calculated from the enrichment analysis; **Holm p** is the *p* value adjusted by Holm-Bonferroni method; the FDR is the *p* value adjusted using False Discovery Rate; **Impact** is the pathway impact value calculated from pathway topology analysis.

## Discussion

3D cell culture methods present a high degree of clinical and biological relevance compared to in vitro models. With the 3D MCS culture, a small aggregate of cells grows and interacts in the 3D microenvironment. The significance of spheroid cultures is increasing rapidly due to advances in microfabricated platforms and allowing large production with consistency^28^. At present, there are several methods to establish the 3D MCS with simplicity and cost-effectiveness. The methods to create 3D spheroids comprise of two systems, including a scaffold-based system and a scaffold-free system^13,29,30^.

Our 3D spheroids were established from CCA and cholangiocyte cell lines by the scaffold-free system, the agarose-coating method (U-surface). This method was adapted from Friedrich et al.^9^, in which the agarose coating on U-surface was performed to create a thin layer with a highly concaved structure on a 96-well plate U-bottom. Our coating technique is based on dynamic agarose coating and aspirating away from the U-bottom 96-well plate. This method provides a very thin gelated layer almost the same shape as the U-bottom plate, which is highly concave. Therefore, this technique is an alternative to the flatbed scheme (non-aspiration). Like other microwell coating methods, this technique is suitable for a variety of treatment conditions to be tested on 3D MCS directly into each well. Since the automated machine recognizes the microplate format, this technique is compatible with both manual coating and automated coating by the automated pipetting system. Moreover, the same plate can be seeded with cells to form spheroids and observed in a high-content automated cell analyzer that recognizes the plate shape and boundaries. Finally, this technique can be used to assemble different cell types to create organoids (e.g., CCA cells and CAFs) with a precise ratio of cell numbers. In addition, a variety of customized agarose-based coating methods have been published, including a rapid and more extensive-scaled generation of 3D MCS^11,12^ and peptide-functionalized agrose^10^ to promote cell attachment and proliferation.

As a result, we successfully developed the 3D spheroid that exhibits a consistent spherical shape and size with reproducibility (1000–50,000 cells per well). The 3D MCSs usually show a microenvironment of cell aggregation, enhanced by the upregulation of cadherins, the cell–cell adhesion molecules^6^. A cholangiocyte cell line (MMNK-1) and cholangiocarcinoma cell lines (KKU-213A and KKU-213C) exhibited the ability to form 3D MCS with upregulations of cell adhesion molecules compared to 2D cultures. However, some cholangiocarcinoma cell lines such as KKU-055 and KKU-100 could not form 3D MCS. That characteristic may relate to their features of poorly-differentiated phenotypes with high expression of vimentin but not cadherin family, which is the epithelial hallmark. This indicates more relation to a mesenchymal phenotype, which is more appropriate for migration rather than cell aggregation^31^.

For cell lines that form 3D MCS, KKU-213A demonstrated the inverse relationship of epithelial-mesenchymal transition (EMT) markers. E- and P-Cadherins were upregulated in this 3D MCS of this cell line, while vimentin was downregulated in 3D MCS. KKU-213C expressed more E-cadherin in 3D MCS but lower in P-cadherin expression. MMNK-1 expressed more N- and P-cadherins in 3D MCS, while no change was observed in vimentin. These results indicated that 3D formation alters the cell–cell junction expressions, which have an influence on vimentin expression (KKU-213A). In the case of non-small-cell lung cancer cell (H460), upregulation or downregulation of E-cadherin were found to increase or decrease 3D spheroid formation, respectively. The results suggested that E-cadherin expression is required for 3D formation^32^. A similar case is also found in pancreatic ductal adenocarcinoma cells (PDAC) which E-cadherin was upregulated in 3D formation of HPAC and BxPC-3 cell lines compared to 2D. Whereas MIA PaCa-2 cells (more mesenchymal phenotype) exhibited the adherens junctions were degraded during EMT and, therefore, not functional and did not form compact 3D spheroids. Further, it could be explained that when the cells are grown in 3D spheroids, the intercellular forces are higher than in the 2D system with higher cell–cell junctions. Whereas in 2D monolayers, the cells encounter less compactness with more cell-substrate focal adhesions. Therefore, cells cultured in these two experimental conditions can experience different forces and, consequently, different mechanical stimulation^33^.

Mostly, cell metabolism studies rely on 2D cultures as they are well established and easy to control in the experimental environment. However, suggestions on improved cell culture systems to reflect the accurate metabolic profiles of tumors had been made^34^. Hence, the 3D cultures are other alternatives to represent extrinsic factors such as nutrient and oxygen gradient, apart from intrinsic genetic factors. Generally, 3D MCS has three zones: an external zone composed of highly proliferating cells; the middle zone of quiescent cells; and an internal zone of necrotic cells^35^. Those layers were influenced by the gradient diffusion of nutrients and oxygen. Although the quiescent zone was not clearly demonstrated in our model, the proliferating and necrotic zones were observed through H&E sections. Also, the unique features of 3D MCSs were corroborated by the presents of hypoxia and oxidative stress observed via the hypoxia probe and oxidative stress probe. Therefore, these 3D MCS models can offer metabolomic alteration and phenotypic diversification regarding nutrient and oxygen proximity, as also demonstrated in the case of gliomas^36^. As a result, It could benefit phenotypic-dependent chemotherapeutic drug screening for CCA.

In this model, the 3D architecture significantly affects the gene expression and promotes energy metabolism alterations of MCSs, especially the glycolytic activity. The rising expression of glucose transporter (GLUT-1) and lactate dehydrogenase A (LDHA) were presented in all 3D MCSs in a similar way to 3D spheroid from human pancreatic ductal adenocarcinoma (PDAC) cell lines^37^. Also, the glycolytic metabolites and their end product, which is lactate, were concurrently accumulated in 3D MCSs. However, the TCA cycle activity was oppositely decreased (TCA metabolites, IDH1, and IDH2), maybe due to the interrupted redox reactions across the cell by low oxygen concentration at the MCS core.

Regarding angiogenesis hallmark, vascular endothelial growth factor C (VEGF-C) was increased in response to this hypoxic condition of hypoxic KKU-055, KKU-213A, and KKU-213C. Lactate has been regarded as an angiogenic factor that accumulates in tumors under hypoxia. It can induce the activation of Raf-ERK pathway and promote angiogenesis and cell growth^38^, and also contribute to the lactate-induced secretion of VEGF, resulting in neoangiogenesis^39^.

Cells are subjected to oxidative stress during the process of 3D formation, and glutathione metabolism plays a role when 3D MCSs try to cope with hypoxic and oxidative stress that takes place inside the structure. Glutathione (GSH) and its precursor, cysteinylglycine, were found to be reduced in 3D MCSs. Our model might be related to in vivo conditions regarding oxidative stress, caused by an imbalance between the ROS and biosynthesis of the antioxidant factors, GSH, that quench them^40^. This model may represent a relevant oxygen gradient condition found in vivo that supports tumor adaptation and drug resistance, as it enforces selective pressure for cancer cells to alter cellular mechanisms that tolerate such conditions^41^.

To date, the study of biomedical research has widely used high-throughput techniques to evaluate the complete set of biomolecules in an organism. Metabolomic analysis, one of the mostly used techniques in systems biology suite, is a powerful tool to study altering metabolisms and metabolites in an organism^42^. Our metabolomic alteration findings reveal that differences between 2D culture and 3D MCS from CCA and cholagiocyte cell lines were determined by the global or untargeted analysis using UHPLC-MS/MS. 3D MCSs of two CCA and a cholangiocyte cell lines exhibited distinct metabolic shifts from their 2D culture systems with cell-specific metabolic signatures. The significant metabolite increase in the glycolytic pathway, including 3-phosphoglycerate, fructose1,6-bisphosphate, and lactate production, correlates with glycolytic enzyme; LDHA high expression in 3D MCSs^43^. Therefore, this pathway may be the additional drug target for CCA with the moderately- and well-differentiated phenotypes (KKU-213C and KKU-213A).

**Figure 7:**
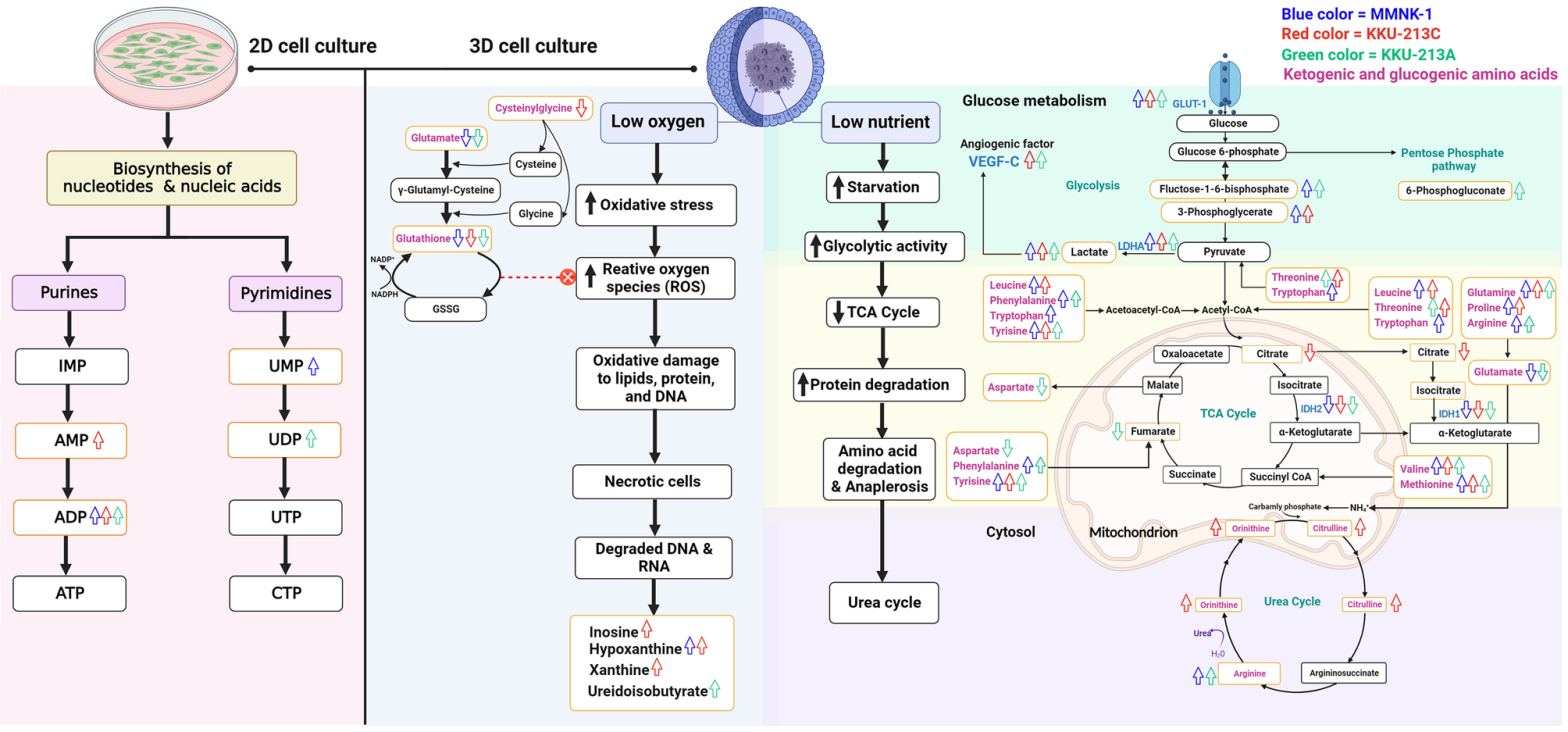
The proposed schematic of influenced pathways in 2D culture and 3D MCS**.** The up and down arrows represent the increased amount and decreased amount, respectively. Metabolites or proteins related to MMNK-1, KKU-213C, and KKU-213A represent in blue, red, and green colors. The illustration was created with BioRender.com.

The uncontrollable proliferation of tumor cells causes progressively hypoxic and oxidative stress conditions. Therefore, tumor cells undergo a series of adaptations that promote the evolution of a more aggressive tumor phenotype, including the activation of DNA damage repair proteins, altered metabolism, and decreased proliferation^44^. However, eukaryotic cells are capable of adaptive responses that reduce the impacts of low O_2_ (hypoxia) to maintain homeostasis. Hypoxia leads to compensatory anaplerosis, whereby glutamine-derived 2-oxoglutarate generated through reductive carboxylation is converted to citrate and subsequently acetyl-CoA, a substrate for lipid synthesis^45^. In our MCS model, there were reductions of TCA intermediates in 3D MCS of some cell lines (fumarate, KKU-213A, and citrate, KKU-213C) but concurrently higher lactate production in all 3D MCSs. Also, the intracellular amino acids, including ketogenic amino acids, are found to be accumulated in 3D MCSs. Hence, there might be protein degradation and anaplerotic reaction taking part to replenish the TCA intermediates. In particular, glutamine may also be metabolized for anaplerosis through glutaminolysis resulting in aspartate and glutamate, which serve as anaplerotic substrates via alpha-ketoglutarate. The proposed metabolic role of anaplerosis is shown in Fig. 7, covering amino acid metabolisms that compensate the insufficient TCA cycle and probably, the electron transport chain (ETC) by converting amino acids into TCA intermediates. In the case of 3D KKU-213C the urea cycle intermediates were also found to be significantly raised, supporting the amino acid degradation process.

3D MCS reflects a more quiescent and stem-like phenotype, while 2D cells represent a more proliferative phenotype. Our metabolomic profile conformed with the high activity of glycolysis and pyruvate metabolism, together with a low activity of the TCA cycle of the patient-derived recurrent-type CCA tissues^46^. There have been evidence that the cancer stem-like cells (CSCs) are involved in cancer recurrence and prefer glycolysis to the TCA cycle to maintain stemness and survival as it can minimize reactive oxygen species (ROS) production^47,48^. 2D cultures, on the contrary, presented with higher intermediates in purines and pyrimidines biosynthesis compared to 3D MCSs. This may relate to the proliferative ability and accelerating production of nucleotides and nucleic acids for cell division.

According to metabolomic results, critical molecular pathways of 3D CCA have been studied in other cancers. The additional drug may be added in combination with the standard regimen to improve the survival outcomes of CCA patients. Currently, the first-line drugs for CCA treatment are gemcitabine and cisplatin, while second-line drugs are FOLFOX (folinic acid, fluorouracil, and oxaliplatin)^49^. These standard drug regimens inhibit mainly DNA synthesis and proliferation of cancer cells. However, targeting other cancer phenotypes may help empower the treatment outcomes. The previous study had shown cisplatin in combination with sulfasalazine (xCT inhibitor) increased drug sensitivity and synergistically suppressed the CD44v9-positive CCA cells (cancer-stem-like phenotype) in vitro and *in vivo*^50^. Therefore, targeting glycolysis, especially lactate production, may be exploited therapeutically for the treatment of CCA. In addition, the LDH inhibitors in combination with gemcitabine were used in pancreatic cancer cells. This synergistic effect is auspicious to attack the key metabolism^51^. Moreover, using the inhibitor to regulate lactate entry or export through the plasma membrane via MCT-4 in 3D MCS was challenging^52^. These schemes may serve as a potential novel regimen for CCA therapeutic strategy by targeting multi-phenotypic CCA cells.

The next-generation 3D model of CCA adds up biological insights for cholangiocarcinoma research and precision medicine. With a 3D microenvironment of CCA micro-tissue, there are many investigations can be carried out, including drug response and penetration in multi-phenotypic CCA spheroids, cancer stem-like cell development, tumor-induced angiogenesis (e.g., co-culturing with endothelial cells), progression of fibrosis (e.g., co-culturing with cancer-associated fibroblasts, CAFs), predicting patient’s drug-response outcomes (constructing from patient-derived cells), immunotherapy model (co-culturing 3D CCA with drug and immune cells)^53,54^. Previously, our group had successfully established the histoculture drug response assay (HDRA) to predict chemosensitivity from patient-derived CCA tissues^55^. The outcomes showed HDRA was an efficient representative model with different drug response patterns. However, the tissue size affects the number of the slice, which influences the replicate number and number of treatment conditions. Also, the slice’s characteristics may differ from region to region of CCA tissue. This MCS formation scheme can also be applied to our ongoing research on engineered-organoid from CCAs and CAFs isolated from patient-derived tissues to yield uniform and quality-controlled scaffold-free CCA organoids. Therefore, the next goal is to evaluate the feasibility of using CCA organoids as a 3D in vitro model to predict patients' response outcomes.

In conclusion, we successfully established a 3D MCS of CCA and cholangiocyte cell lines with an easy and convenient scheme. The MCSs showed uniformity with quality controllability in size and shape suitable for high content drug screenings. The MCSs also presented a unique hypoxic and oxidative stress microenvironment that affects alterations in their metabolisms. Proteo-metabolomic results indicated 2D cultures are prone to proliferative phenotypes while 3D MCSs are more similar to quiescent and stem-like phenotypes. Increased activity of glycolysis, amino acid degradation, and anaplerotic reactions to compensate for the TCA cycle and OXPHOS were observed. Our results elucidate the proteo-metabolomic changes in 3D MCSs. These findings may be a powerful tool for novel combinatory-drug studies and the development of next-generation CCA organoids that help predict the CCA treatment outcomes.

## Limitations of this study

The investigation of 3D MCSs in this study relies on bulk analyses. However, it is noted that the outer layer of 3D MCS may still be proliferative phenotype as the cells are directly in contact with oxygen and nutrients. To confirm the phenotypes across different zones of MCS, recently available spatial analyses, including spatial transcriptomics or spatial proteomics, may help elucidate better phenotypic and zonal visualization of MCS sections. Also, targeting additional biological pathways is still an important consideration. Hypoxia-inducible factor 1 (HIF-1) is potentially coming to attention as it is the master regulator of cellular oxygen sensing, which relates to the activation of downstream survival genes. Lactate dehydrogenase, as well as MCT-4 facilitates lactate excretion, are also interesting targets. Finally, the metabolomic study excluded the CCA cell lines with poorly-differentiated phenotypes (KKU-055 and KKU-100) as they did not form a 3D structure in the scaffold-free condition. The following proteo-metabolomic investigations would use the scaffold-based (*e.g*. Matrigel®) to assist the formation of 3D KKU-055 and KKU-100 in the artificial ECM.

## Methods

### Cell lines

All cholangiocarcinoma cell lines (KKU-055, KKU-100, KKU213C, and KKU-213A) and immortalized cholangiocyte cell lines (MMNK-1) were purchased from the Japanese Collection of Research Bioresources (JCRB) Cell Bank.

### Cholangiocarcinoma and cholangiocyte cultures

MMNK-1, immortalized cholangiocyte cell line was maintained in HAM’s F-12 Medium supplemented with 10% FBS and 1% penicillin/streptomycin (Thermo Fischer Scientific, Massachusetts, USA). The cholangiocarcinoma cell lines, including KKU-055, KKU-100, KKU-213C, and KKU-213A, were maintained in Dulbecco's modified Eagle's medium (DMEM) supplemented with 10% FBS and 1% penicillin/streptomycin (Thermo Fischer Scientific, Massachusetts, USA). Subcultures were performed every 3 days. All cell lines were cultured at 37 °C, 95% humidity, and 5% CO_2_ condition.

### 3D MCS culture of cholangiocarcinoma and cholangiocyte cell lines

Agarose (Molecular Biology Grade, GENEDIREX, Inc.) was dissolved in phosphate-buffered saline (PBS) 1X, pH 7.4 (Thermo Fisher Scientific, Massachusetts, USA), and autoclaved. Before coating, agarose gel was molten by a microwave oven. Then, 200 ul of liquid agarose was dispensed into the well of the U-bottom 96-well plate (Corning® 96-well Clear Polystyrene Microplates), and all amount was quickly aspirated away. This will leave an instant thin-layer coating along the U-shape well. The cells were seeded into each well straight away after plate coating, without centrifugation, and allowed 2 days of culture at 37 °C, 95% humidity, and 5% CO_2_.

### 3D MCS quality control

On a routine basis, cell and spheroid images were captured using a phase-contrast microscope (Axiovert 40 inverted microscope, ZEISS) at 4X and 10X objective lens via program (iSolution Lite, IMT i-Solution Inc.) For MCSs, the images were measured with the diameter and projection area using ImageJ (the Research Services Branch, National Institute of Mental Health, Bethesda, Maryland, USA.). The MCS size, which was deviated more than 10% of the average, was excluded from the experiment.

### Assessment of 3D MCS layers

MCSs were fixed with 4% paraformaldehyde and rinsed with PBS. Then, the MCSs were dehydrated using a series of ethanol (70%, 80%, 95%, and absolute ethanol). After that, samples were exchanged with xylene and paraffin. According to standard protocols, the MCSs were embedded in paraffin, sectioned, and stained with hematoxylin and eosin (H&E). Then, the sections were visualized by light microscopy (fluorescence imaging microscope/NiU), and images were obtained with a digital camera (NIS-Element D 4.00.00.lnk).

### Assessment of hypoxia and oxidative stress of spheroid formation

Hypoxic condition and oxidative stress of MCSs were detected via ROS-ID® Hypoxia/Oxidative stress detection kit (Enzo Life Sciences, Inc., New York, USA). Briefly, live MCSs were formed in culture media containing hypoxia (red) and oxidative stress (green) fluorescent probes and incubated at 95% humidity and 5% CO_2_ condition. The generated fluorescent products can be visualized using a wide-field fluorescent microscope equipped with standard fluorescein (490/525 nm) and Texas Red (596/670 nm) filters.

### Assessment of protein expression via western blot analysis

The cells of 2D cultures and 3D MCSs were harvested as pellets by centrifugation and washed with PBS. The whole-cell lysates were prepared by lysing pellets in RIPA buffer (150 mM NaCl, 50 mM Tris–HCL pH 7.4 (4 °C), 1% Tween-20 in TritonX, 1% sodium-deoxycholate, 10% SDS) containing protease inhibitor cocktail (Roche, Basel, Switzerland). Protein concentrations were evaluated with Pierce™ BCA Protein Assay Kit. The extracted proteins (20 µg) were mixed with loading dye and separated on SDS-PAGE. After that, they were transferred to a polyvinylidene difluoride membrane (Whatman, Dassel, Germany) under 250 mA, free voltage for 1.40 h. When finished transferring, the membranes were cut according to the prestained protein ladder (ab116027, Abcam, UK ) to cover the target band. The pre-cut membranes were blocked with 5% skim milk and probed with primary antibodies at 4 °C overnight. On the next day, the membranes were washed and probed with horseradish-peroxidase-conjugated secondary antibodies at room temperature for 1 h. Signals were detected using chemiluminescence ECL (Amersham, Italy). The list of primary and secondary antibodies used in this experiment were listed in supplementary Table S2.

### The global metabolomic analysis

#### Sample preparation for UHPLC-Q-TOF-MS/MS

Three independent experiments of cholangiocarcinoma and immortalized cholangiocyte cell lines (2D culture and 3D MCS) were cultured in 37 °C, 95% humidity, 5% CO_2_ condition, and harvest cell pellets. The cells were trypsinized and collect at the concentration of 1 × 10^6^ cells. The cell pellets were washed with phosphate-buffered saline (PBS), pH 7.4 (Thermo Fisher Scientific, Massachusetts, USA) 2 times. The last centrifugation was performed at 5,000 rpm, 5 min 4 °C, and discard the supernatant. Next, methanol (HPLC grade) was added to each sample tube and dipped into liquid nitrogen until freezing. Then, the tubes were thawed in an ice bath and sonicated. Phase extraction was performed by adding with cold water (HPLC grade) and chloroform which made up of methanol: water: chloroform to 1:1:3. incubate on ice and centrifuge 4000 rpm, 20 min at 4 °C and then transfer each phase (Aqueous phase, Proteins and Macromolecule, Organic phase) into tube. Aqueous phase remove solvent by vacuum concentrator. Organic phase remove solvent by dried in hood. Dissolve sample in reconstitution buffer and transfer to vial for LC–MS data acquisition.

#### Data acquisition

Metabolomic analysis was carried out by the Khon Kaen University Phenome Centre (KKUPC) using ultra-high performance liquid chromatography coupled with electrospray ionization-quadrupole time-of-flight mass spectrometry (compact UHPLC ESI-Q-TOF MS, Bruker, DE). In brief, the aqueous phase extracts of samples were analyzed in reverse-phase liquid chromatography platform. The samples were injected into the UHPLC system equipped with a Bruker intensity C18 (2.1 mm × 100, 1.9 um). The temperature of the column was set at 40 °C, and the temperature of the autosampler was set at 4 °C. Mobile phase A comprised of water 100% mixed with 0.1% formic acid (FA), and mobile phase B is comprised of acetonitrile 100% mixed with 0.1% FA. The flow rate was set at 0.35 ml/min, and the elution gradient was set at 99% A (0.0–2.0 min, 0.25 ml/min), 99–1% A (2.0–17.0 min, 0.25 ml/min), 1% A (17.0–20.0 min, 0.25 ml/min), 1–99% A (22.0–30.0 min, 035 ml/min). An injection volume of sample 7 ul was applied in both positive and negative ionization polarity mode. The MS system was set temperature 220 °C, desolvation gas 8 L/min. Sodium formate (2 mM sodium hydroxide, 0.1%FA and 50% IPA) was injected with calibrant at flow rate 0.4 ul/min. The ionization voltage in positive and negative modes were 4000 and 4500 V, respectively. The data scan range was set mass range 50–1500 m/z). For QC sample was injected 15 times before initiating the actual batch run for conditioning the column. The QC sample was reinjected with the interval of four samples, and at the end, to monitor the instrument stability and analyze reproducibility.

### Data processing

All spectra were imported into MetaboScape4.0 software (Bruker, Massachusetts, US) for data processing. The untargeted metabolic analysis was identified using through mass-based search with the mass to charge ratio (m/z) and retention time of a molecular ion, which is searched against the database(s), e.g., Human Metabolome Database (HMDB) (https://hmdb.ca), Metlin (https://metlin.scripps.edu). The datasets mass intensity of interested metabolites were interpreted with pathway analysis and hierarchical clustering using MetaboAnalyst 5.0 (https://www.metaboanalyst.ca/)^21^. Then we construct the metabolic reaction networks using MATLAB R2015a (The MathWorks, Inc.) software equipped with MetaboNetwork toolbox^23^ and the background shading was added to the graphs to indicate the different interconnecting pathways^27^.

### Statistical analysis

#### Data representation and statistical analysis

The student’s t-test was used to analyze differences between two groups, and Two-way ANOVA was used to analyze intergroup differences between two groups*. P*-values less than 0.05 were considered statistically significant. The analysis was performed using GraphPad Prism 9.3.1 (GraphPad Software). Densitometry results of Western Blots were quantified using ImageJ software. All data are presented as mean and SEM, and other details such as the number of replicates and the level of significance are mentioned in figure legends and supplementary tables.

For multivariate analysis, the datasets were circulated for Principal component analysis (PCA) and orthogonal projection to latent structures-discriminant analysis (OPLS-DA) using the two-difference application, namely MATLAB R2015a (The MathWorks, Inc.) and SIMCA-14.1 (Umetrics, Sartorius Stedim Biotech). The unsupervised method, PCA, can be used to visualize the possible related principle component, in terms of data variations, by minimizing the data dimensions of the huge dataset. As a matter of fact, this unsupervised PCA method can be applied to distinguish the intrinsic similarities or differences of the data. On the other hand, the supervised OPLS-DA method detailed the typical metabolite, which exhibited the difference beyond the class^7^.

## Supplementary Information

Supplementary Information 1.

Supplementary Information 2.

## Data Availability

The datasets generated and analyzed during the current study are available in the OSF repository, https://osf.io/t25yv. Raw data of metabolic acquisition of 2D cultures and 3D MCSs from UHPLC-MS/MS (.csv files) were uploaded (https://doi.org/10.17605/OSF.IO/T25YV).The results generated from the comparative analyses supporting the findings of this study are available within the paper and its supplementary information.
