# Supplementary material for: The impact of hypoxia and oxidative stress on proteo-metabolomic alterations of 3D cholangiocarcinoma models
Source: Sci Rep. 2023 Feb 21;13:3072. doi: 10.1038/s41598-023-30204-y (PMC9944917; doi:10.1038/s41598-023-30204-y)
Supplement: Supplementary file 2 — Supplementary Information 2. [file 41598_2023_30204_MOESM2_ESM.docx]

The impact of hypoxia and oxidative stress on proteo-metabolomic alterations of 3D cholangiocarcinoma models

Pimpawadee Phukhum^1,3^, Jutarop Phetcharaburanin^1,2,3^, Kwuanjira Chaleekarn^1,3^, Yingpinyapat Kittirat^1,3^, Thanaporn Kulthawatsiri^2,3^, Nisana Namwat^1,2,3^, Watcharin Loilome^1,2,3^, Narong Khuntikeo^2,3,4^, Attapol Titapun^2,3,4^, Arporn Wangwiwatsin^1,2,3^, Tueanjit Khampitak^1^, Manida Suksawat^2,3^, Poramate Klanrit *^,1,2,3^

^1^Department of Biochemistry, Faculty of Medicine, Khon Kaen University, Khon Kaen 40002, Thailand

^2^Khon Kaen University International Phenome Laboratory, Khon Kaen, 40002, Thailand

^3^Cholangiocarcinoma Research Institute, Khon Kaen University, Khon Kaen 40002, Thailand

^4^Department of Surgery, Faculty of Medicine, Khon Kaen University, Khon Kaen 40002, Thailand

*Corresponding Author: [porakl@kku.ac.th](mailto:porakl@kku.ac.th)

**Supplementary_material**

**Table S1** CCA and cholangiocyte cell lines and characteristics

| **Cell line** | **Source** | **Profile** | **References** |
| --- | --- | --- | --- |
| MMNK-1 | JCRB Cell Bank* | Highly differentiated immortalized human cholangiocyte cell line | Maruyama et al., 2004 |
| KKU-100 | JCRB Cell Bank | Human poorly differentiated cholangiocarcinoma cell line established from biliary tract | Sripa et al., 2005 |
| KKU-055 | JCRB Cell Bank | Human poorly differentiated cholangiocarcinoma cell line established from biliary tract | Sripa et al., 2005 |
| KKU-213A (previous KKU-213) | JCRB Cell Bank | Human adenosquamous cell carcinoma and well-differentiated cell line established from biliary tract | Sripa et al., 2020 |
| KKU-213C (previous KKU-156) | JCRB Cell Bank | Human moderately differentiated adenocarcinoma cell line established from biliary tract | Sripa et al., 2020 |

***Japanese Collection of Research Bioresources Cell Bank, Osaka, Japan**

**Table S2** List of primary and secondary antibodies

| ANTIBODY | SOURCE | IDENTIFIER |
| --- | --- | --- |
| Anti-E-Cadherin  Anti-N-Cadherin  Anti-P-cadherin  Anti-Vimentin  Anti-β-Actin  Anti-GLUT-1  Anti-GAPDH  Anti-LDHA  Anti-IDH1  Anti-IDH2  Anti-VEGF-C  Secondary Anti-Rabbit  Secondary Anti-Mouse | BD Bioscience  Thermo Fisher Scientific  Thermo Fisher Scientific  Abcam  SIGMA  Thermo Fisher Scientific  Thermo Fisher Scientific  Thermo Fisher Scientific  Thermo Fisher Scientific  Thermo Fisher Scientific  Abcam  Dako EnVision  Dako EnVision | Cat# 610182  Cat# 33-3900  Cat# 32-4000  Cat# ab92547  Cat# A5441  Cat# PA1-46152  Cat# MA5-15738  Cat# PA5-27406  Cat# MA5-27759  Cat# PA5-79436  Cat# ab135506  Cat# K4003  Cat# K4001 |

**
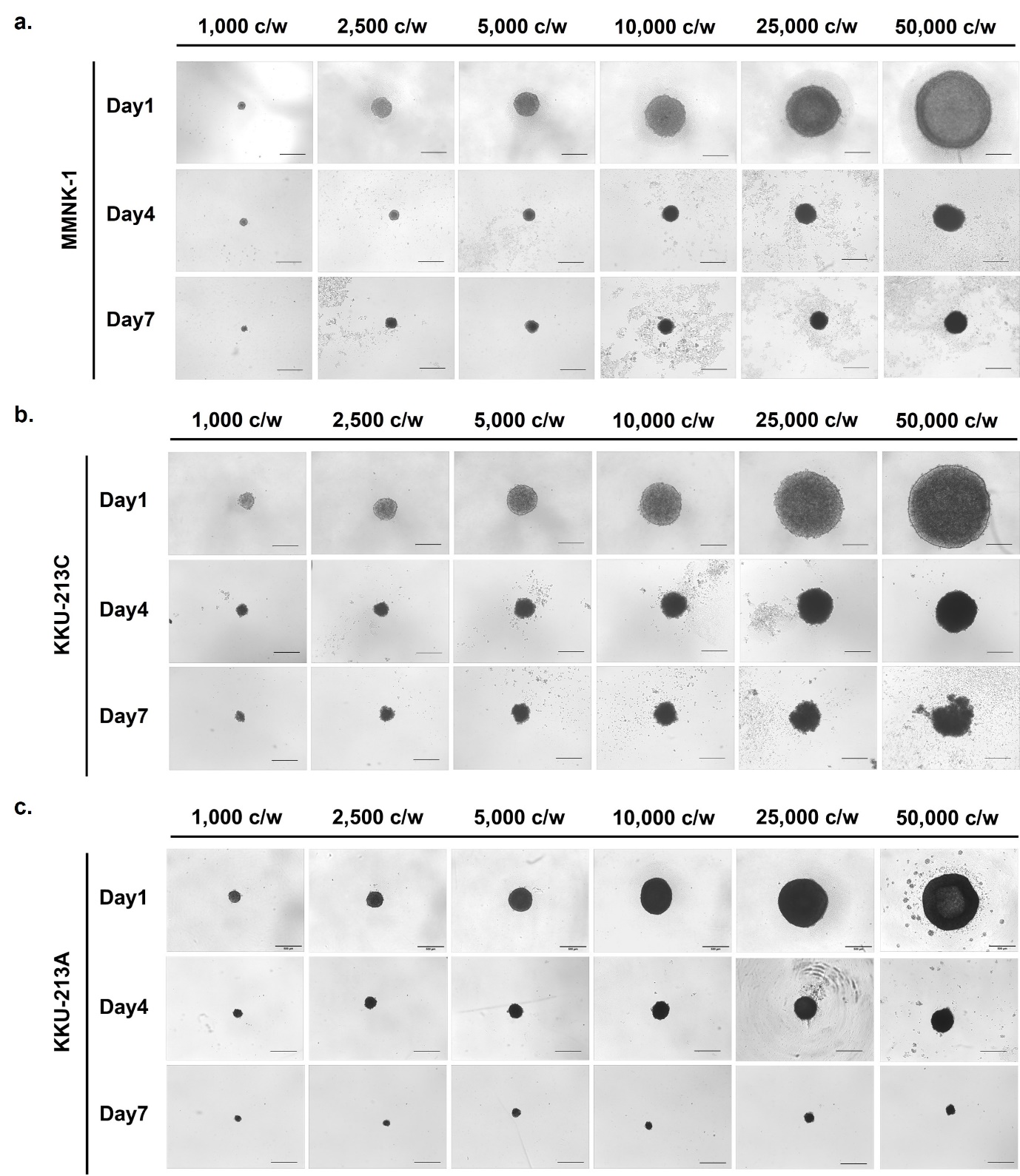
**

**Figure S1. Characterization MCS morphology.** CCA and cholangiocyte cell lines were varying cell-seeded numbers (1,000 2,500 5,000 10,000 25,000 and 50,000 cells/well) and cultured for 1-7 days to observe the MCSs aggregation under an inverted microscope.
(A) MMNK-1, 1,000-50,000 cells/well and cultured for 1-7 days.
(B) KKU-213C, 1,000-50,000 cells/well and cultured for 1-7 days.
(C) KKU-213A, 1,000-50,000 cells/well and cultured for 1-7 days.


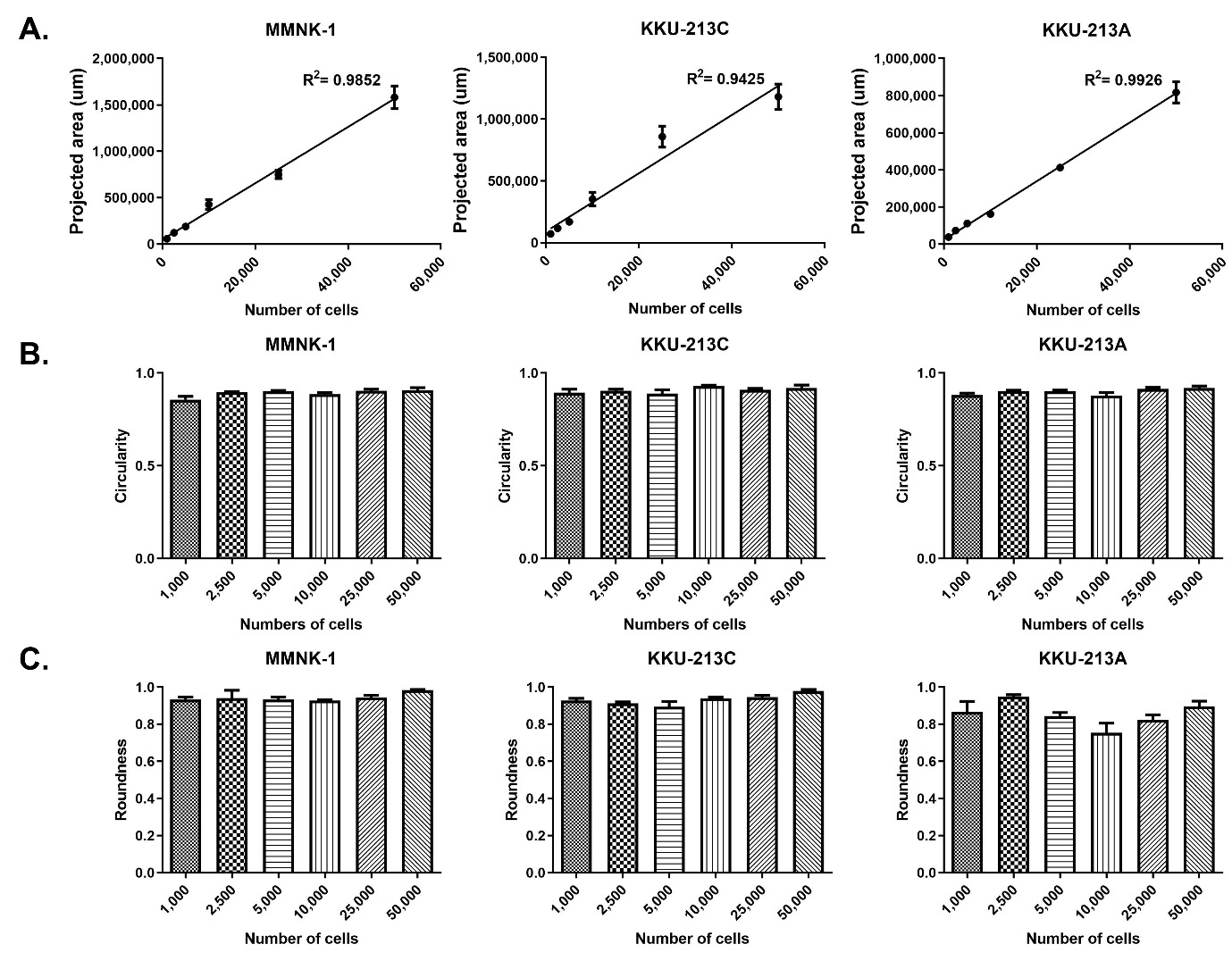


**Figure S2** **The 3D MCSs of CCA and cholangiocyte cell lines optimization**(A) The projected area of 3D MCSs post-seeding of 1,000-50,000 cells/well.
(B) The circularity of 3D MCSs of CCA post-seeding of 1,000-50,000 cells/well.
(C) The roundness of 3D MCSs of CCA post-seeding of 1,000-50,000 cells/well.


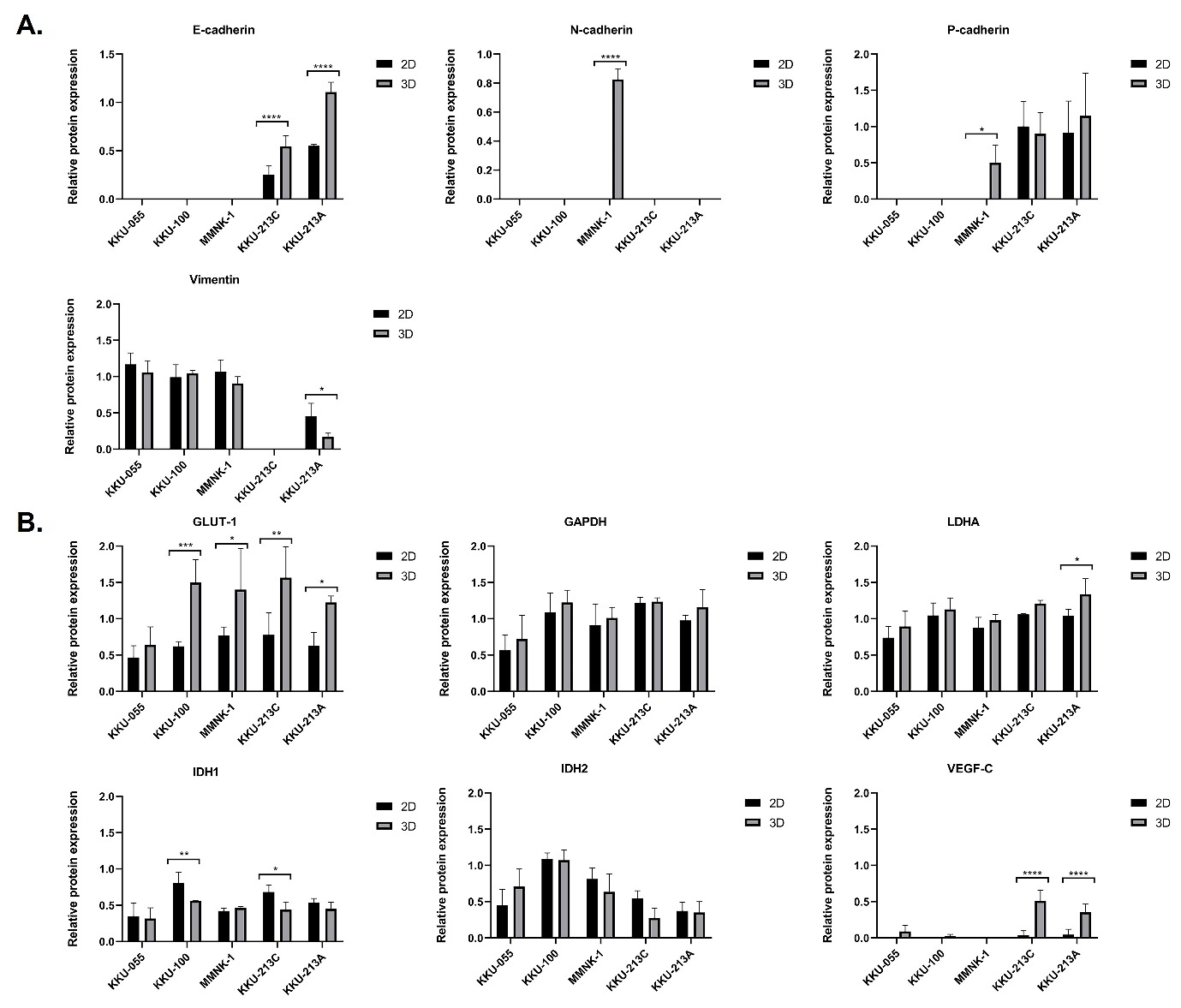


**Figure S3 The protein expression of 2D vs. 3D CCA and cholangiocyte cell lines via western blot analysis.**(A) western blot analysis of cell adhesion molecules (E-cadherin, N-cadherin, and P-cadherin) and vimentin.
(B) A protein and enzymes involved in the glycolytic pathway (GLUT-1, GAPDH, and LDHA), enzymes related to TCA cycle (IDH1 and IDH2) and angiogenic activity (VEGF-C) express on 2D and 3D of CCA and cholangiocyte cell lines. Quantitative analysis of protein (normalized to β-actin). Data are means ± S.D. of at least three independent experiments. **P*< 0.05, ***P*< 0.01 and *****P*< 0.01.


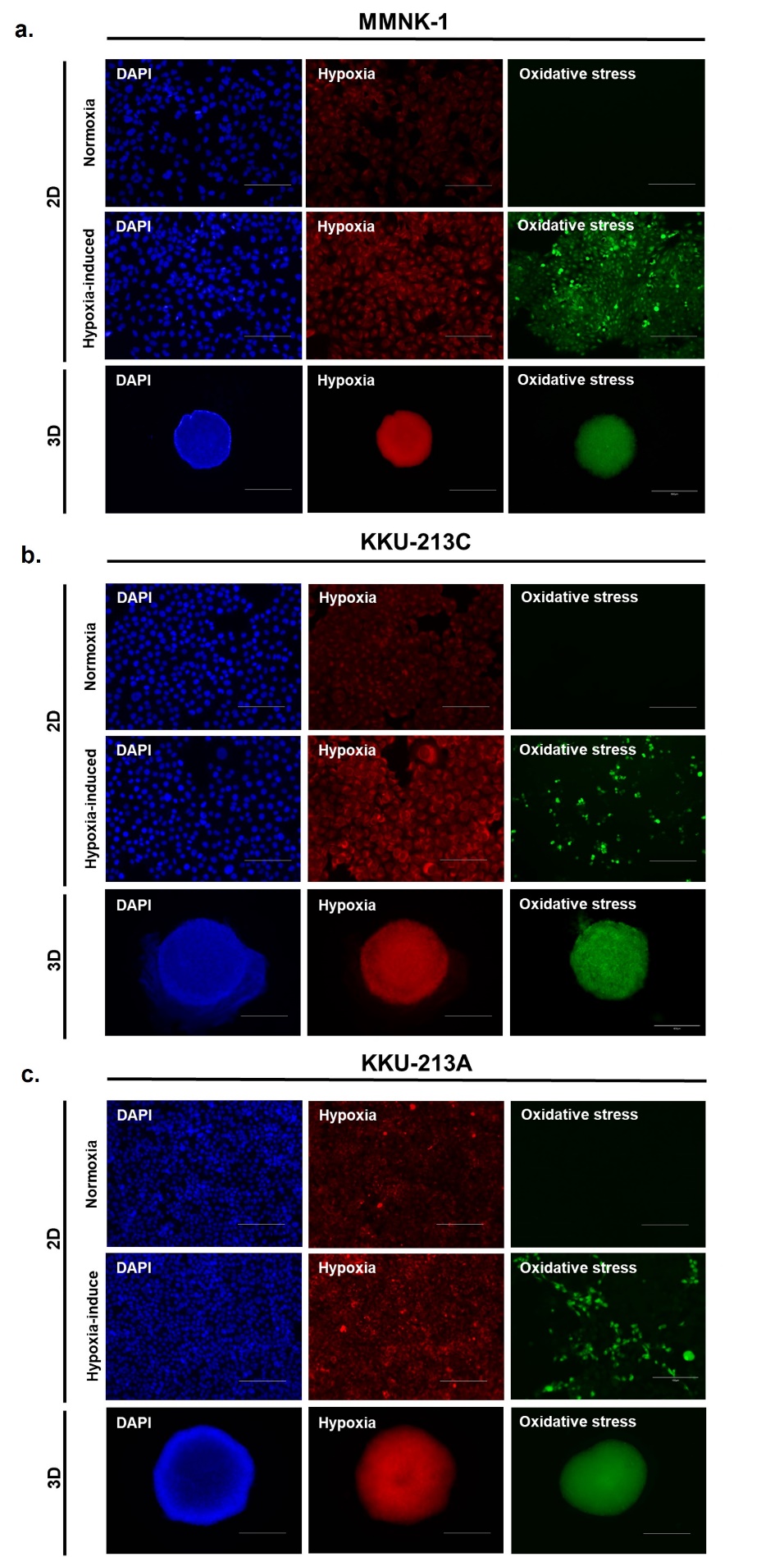


**Figure S4. Hypoxia and oxidative stress in 2D culture vs. 3D MCS .** The 3D MCS exhibit typical zonation of hypoxia and oxidative stress. DAPI (blue), Hypoxia (red), Oxidative stress (green) and the last panel (merge). Scale bar represents 800 µm**.** (A) MMNK-1 exhibit typical zonation of hypoxia and oxidative stress. (B) KKU-213C exhibit typical zonation of hypoxia and oxidative stress. (C) KKU-213A exhibit typical zonation of hypoxia and oxidative stress.


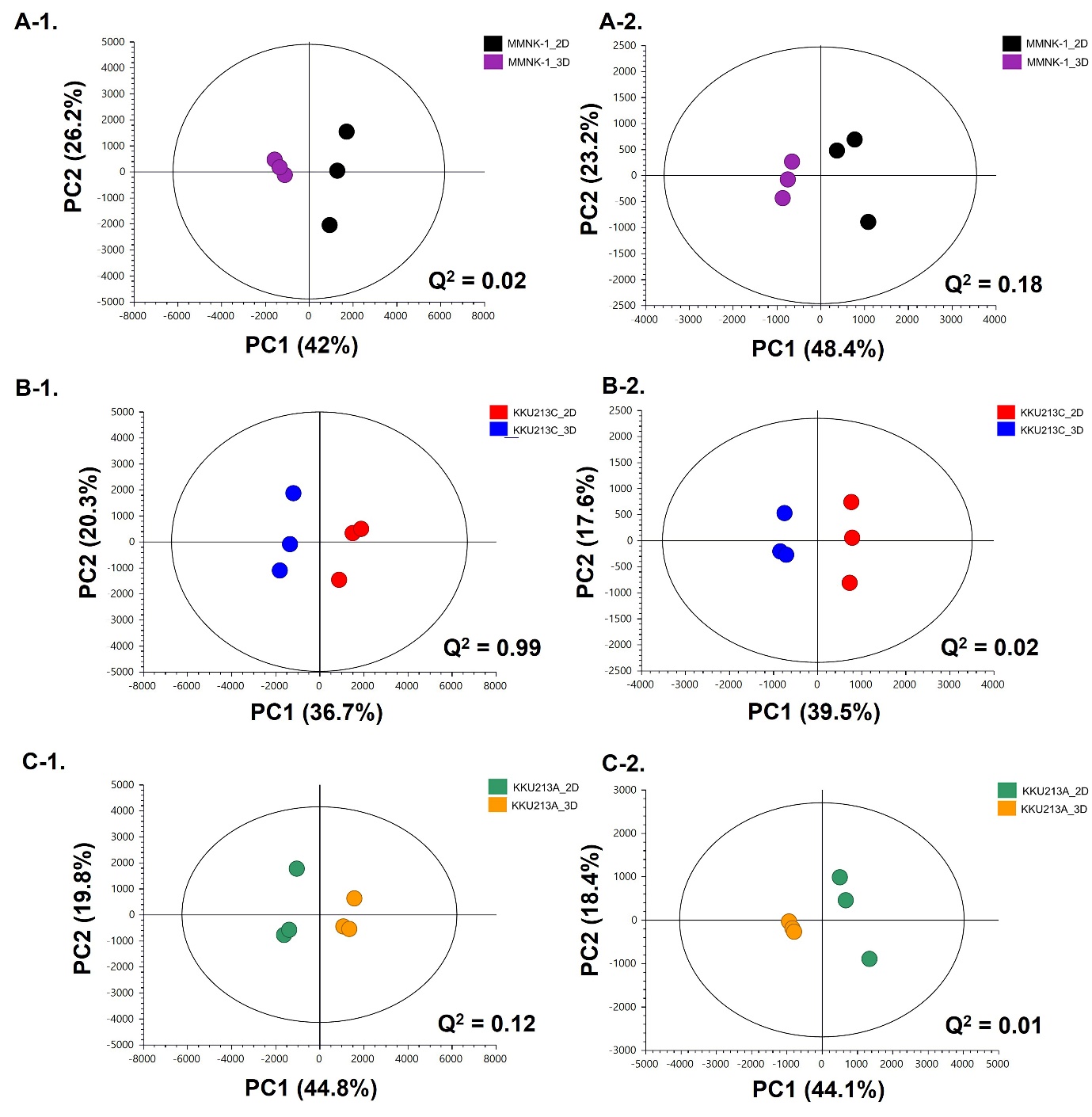


**Figure S5.** **PCA score plots** base on UHPLC-ESI-Q-TOF-MS/MS data set of 2D and 3D form CCA cell lines (A), MMNK-1-2D (black) and MMNK-1-3D (purple); (B), KKU-213C-2D (red) and KKU-213AC-3D (blue); (C), KKU-213A-2D (green) and KKU-213A-3D (yellow). Left and right panels represent ESI+ ionization mode (1) and ESI- ionization mode (2).


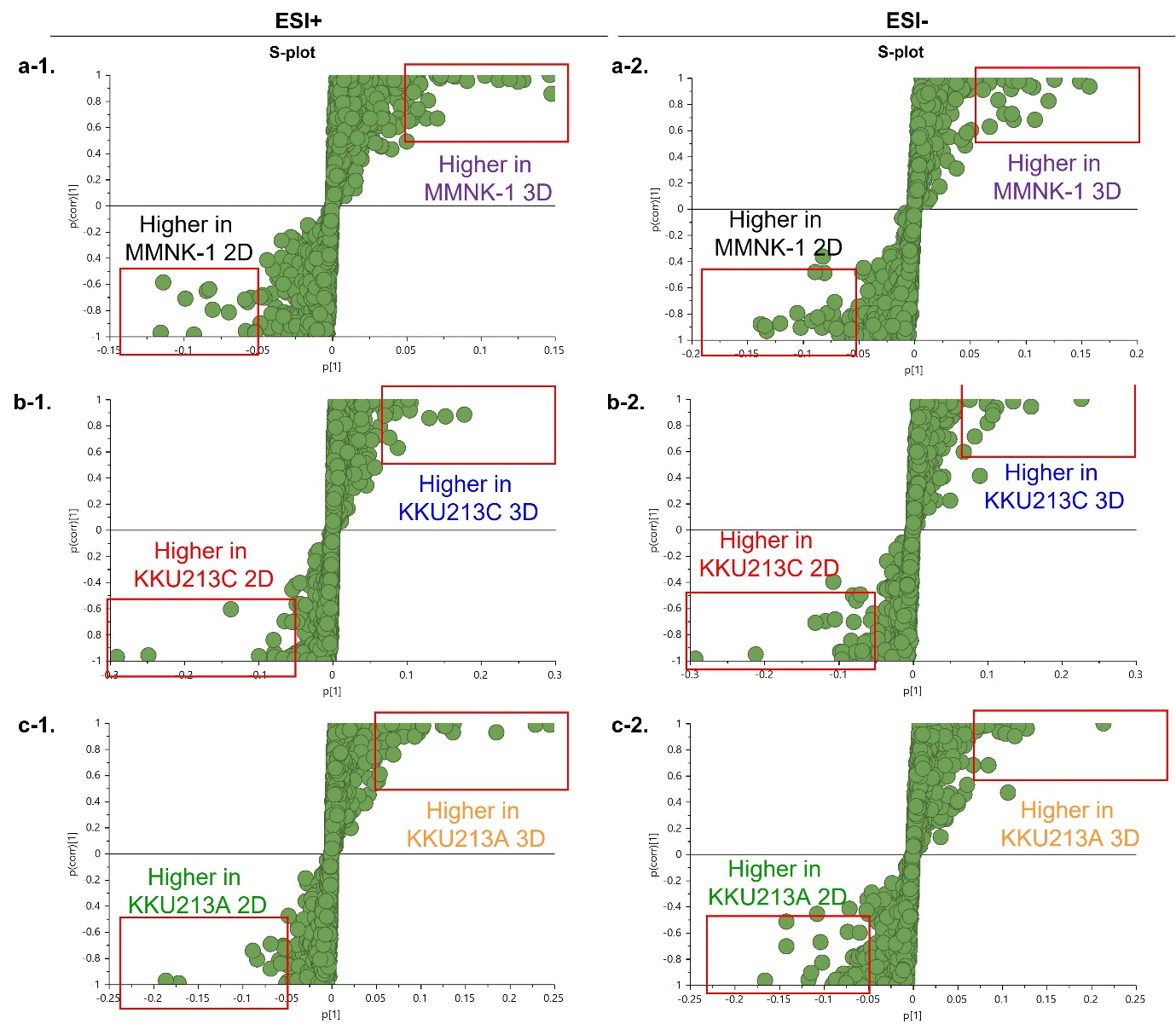


**Figure S6 S-plot** base on UHPLC-ESI-Q-TOF-MS/MS data set of 2D and 3D form CCA cell lines (a), MMNK-1-2D (black) and MMNK-1-3D (purple); (b), KKU-213C-2D (red) and KKU-213AC-3D (blue); (c), KKU-213A-2D (green) and KKU-213A-3D (yellow). Left and right panels represent ESI+ ionization mode (1) and ESI- ionization mode (2).


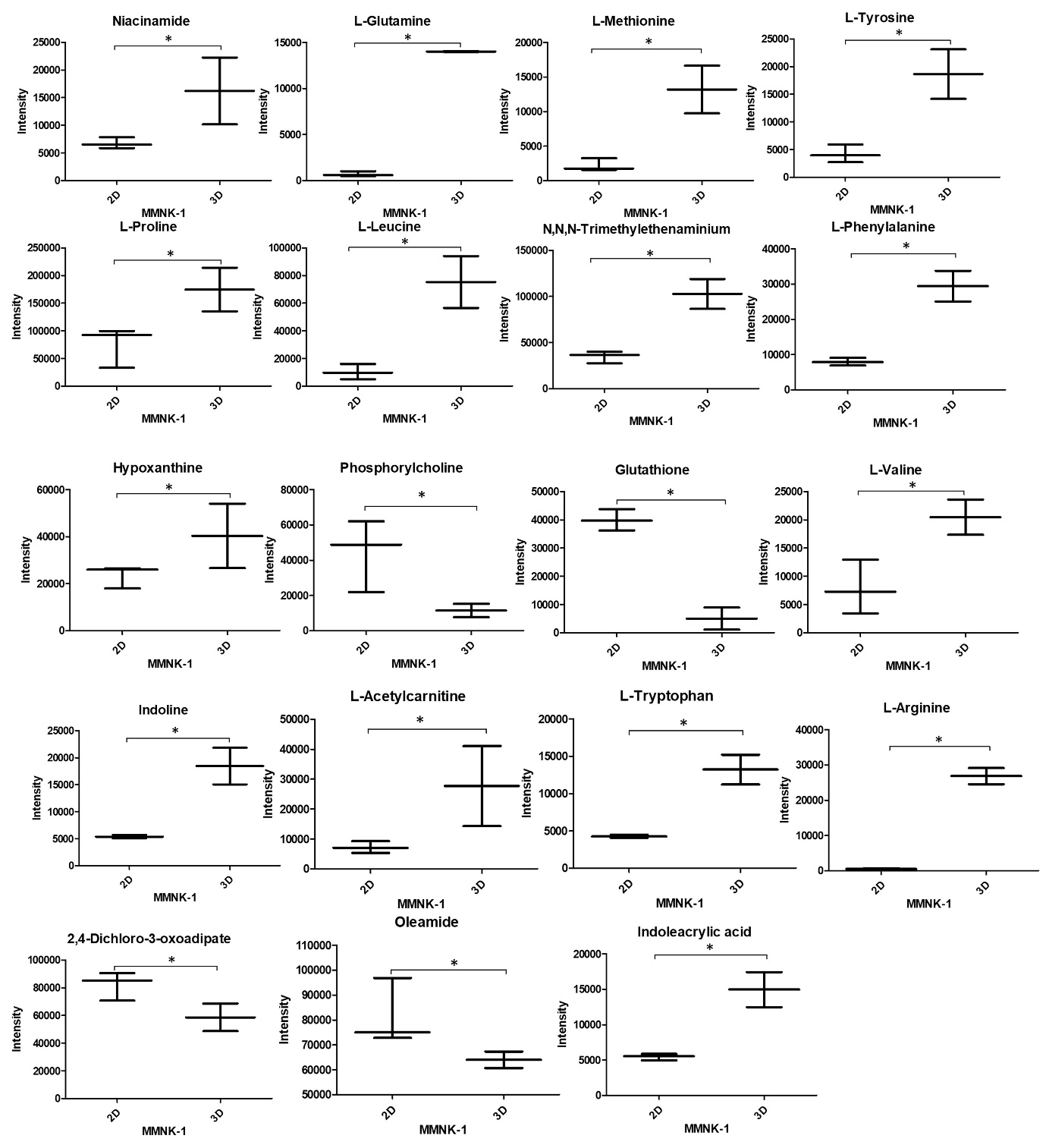


**Figure S7.** Intensity of candidate metabolites of MMNK-1 (2D VS 3D) in positive mode.

Statistically significant was determine as * indicate p-value <0.05.


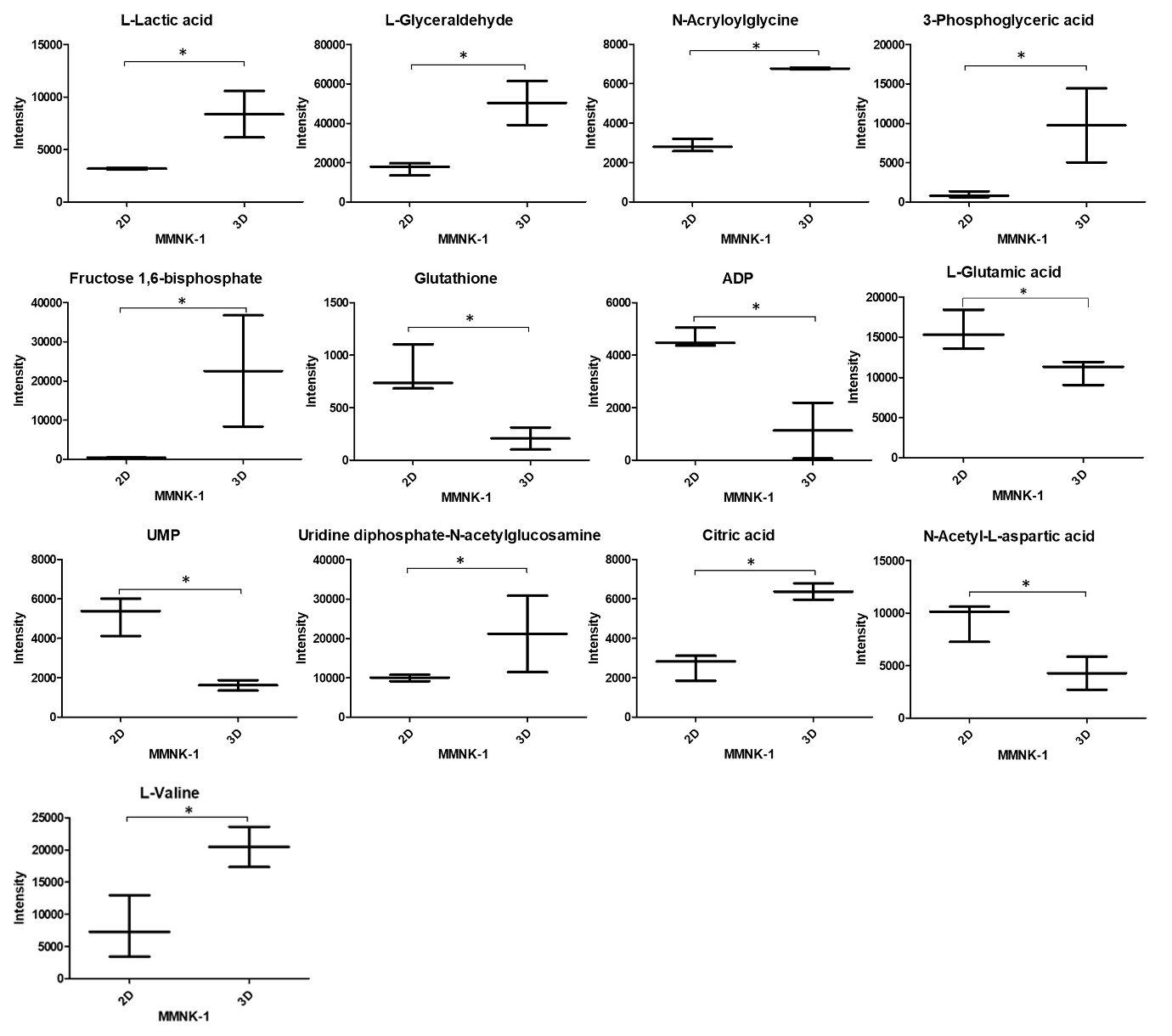


**Figure S8.** Intensity of candidate metabolites of MMNK-1 (2D VS 3D) in negative mode.

Statistically significant was determine as * indicate p-value <0.05.


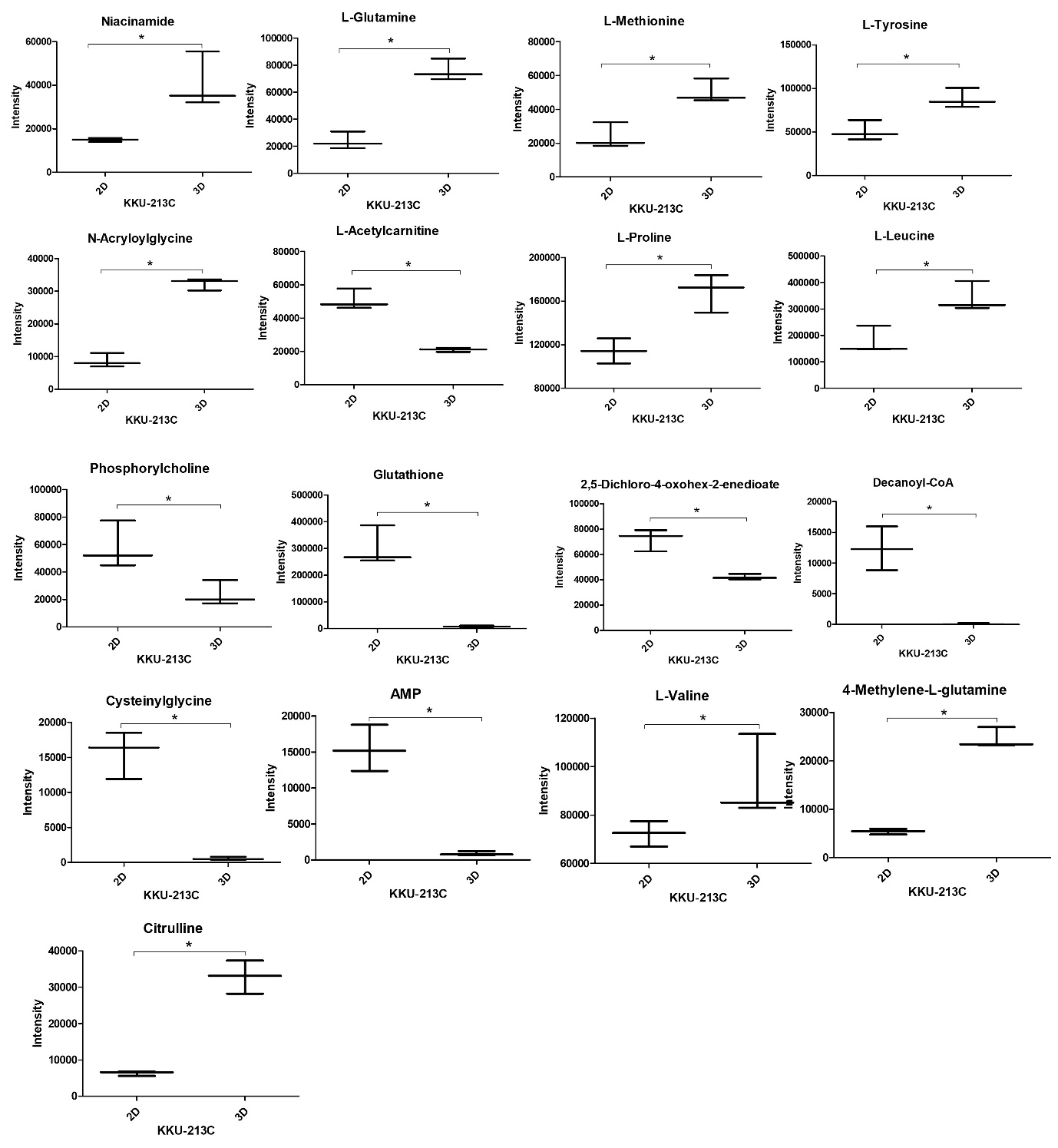


**Figure S9.** Intensity of candidate metabolites of KKU-213C (2D VS 3D) in positive mode. Statistically significant was determine as * indicate p-value <0.05.


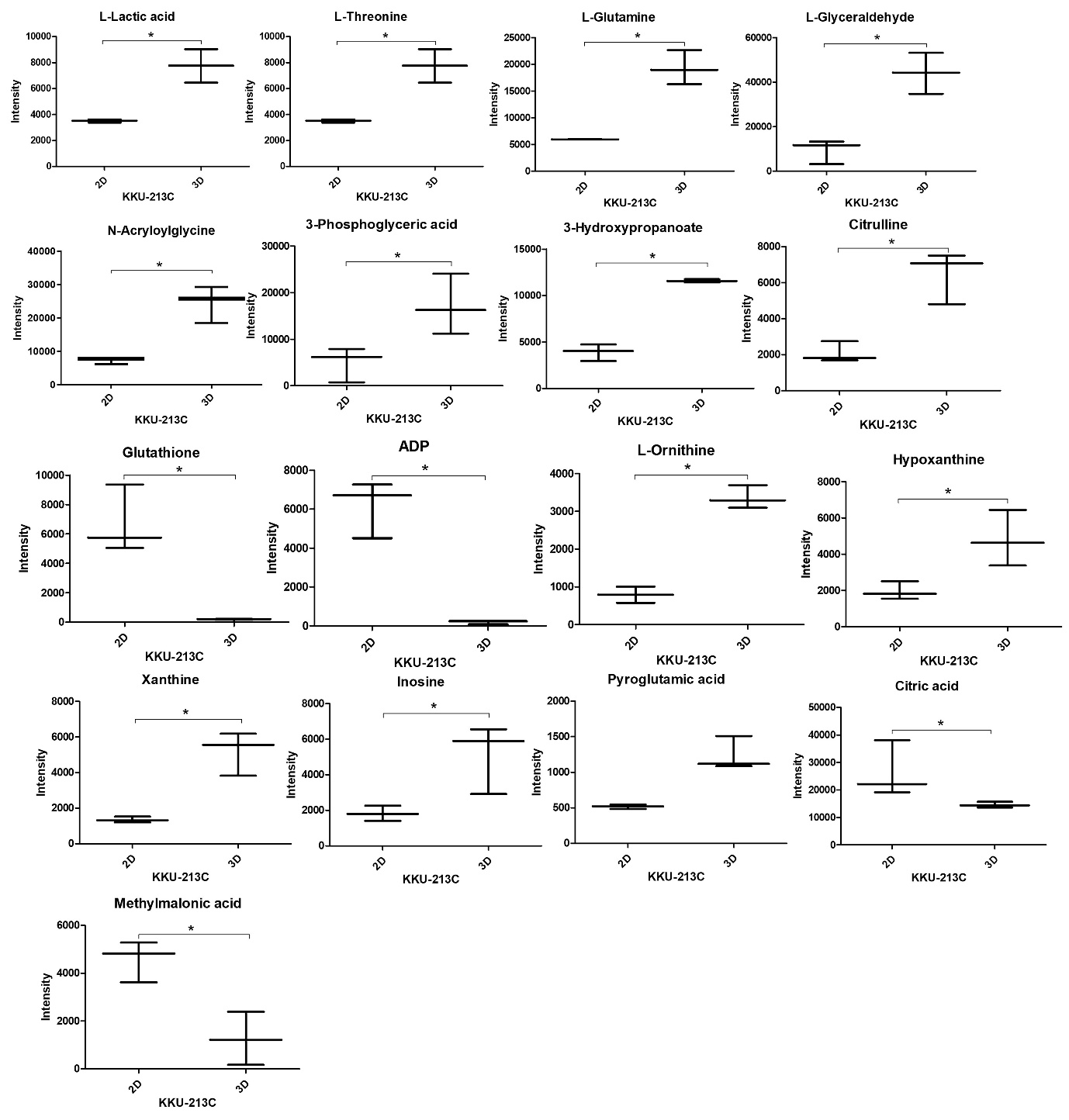


**Figure S10.** Intensity of candidate metabolites of KKU-213C (2D VS 3D) in negative mode. Statistically significant was determine as * indicate p-value <0.05.


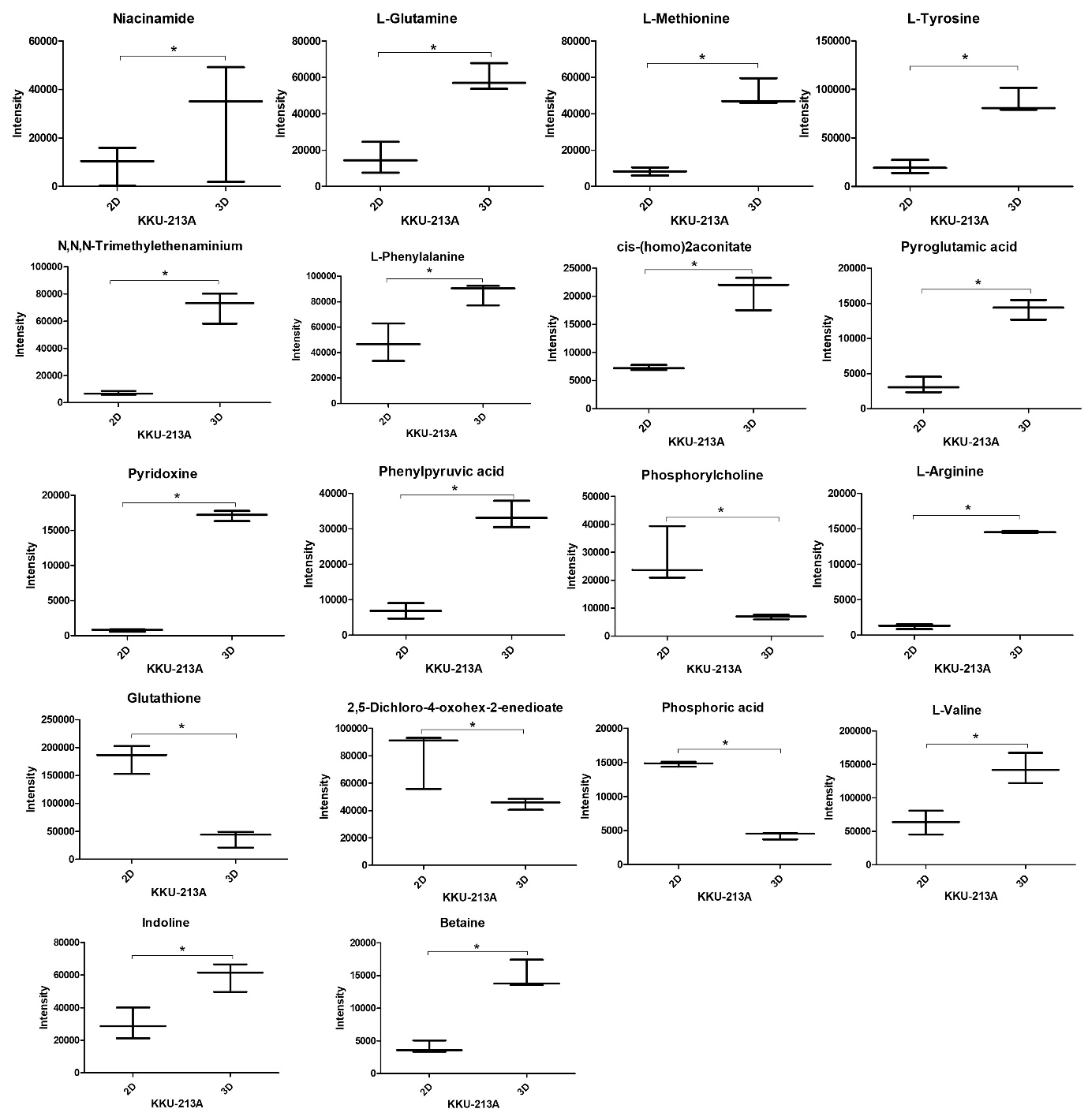


**Figure S11.** Intensity of candidate metabolites of KKU-213A (2D VS 3D) in positive mode. Statistically significant was determine as * indicate p-value <0.05.


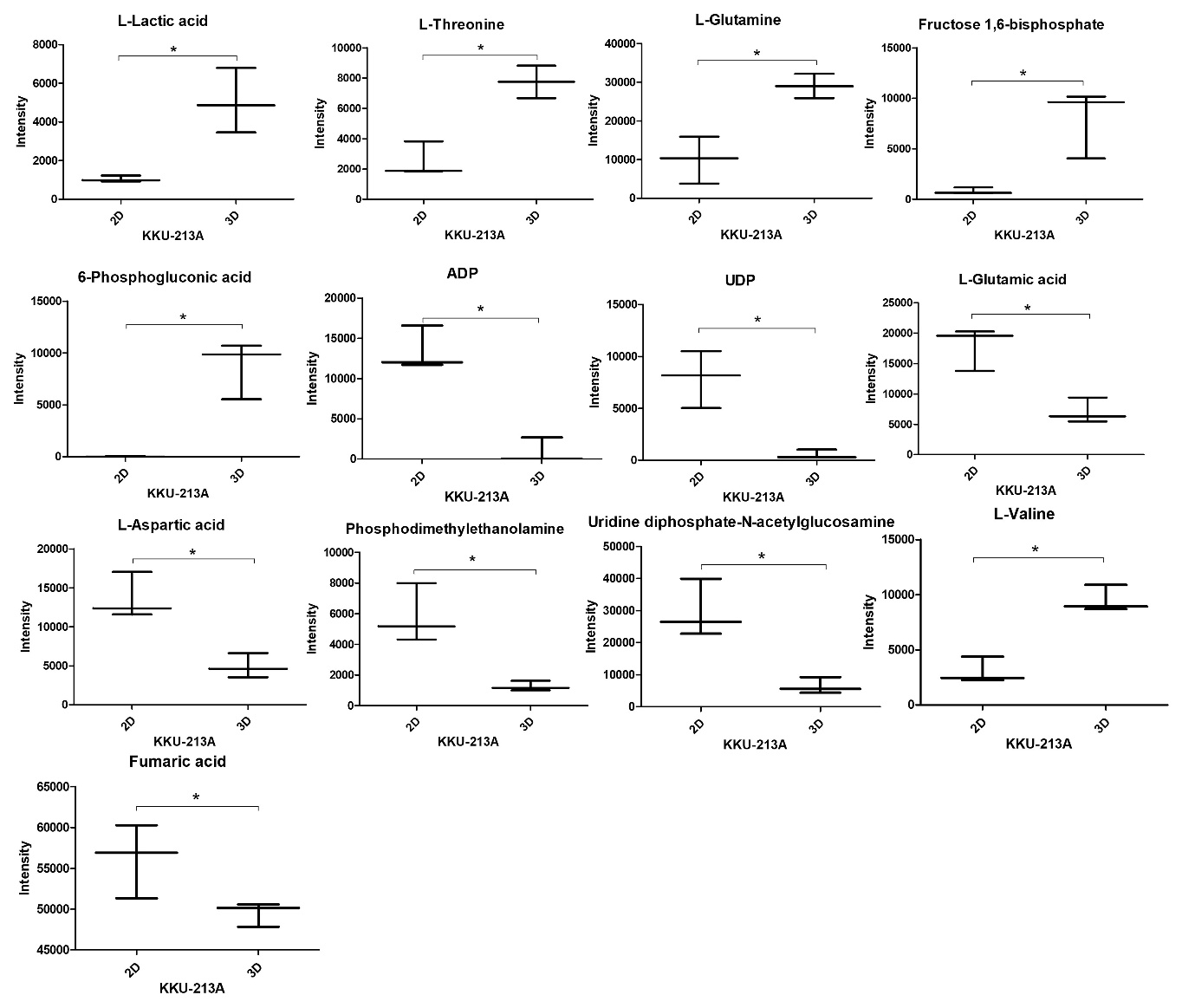


**Figure S12.** Intensity of candidate metabolites of KKU-213A (2D VS 3D) in negative mode. Statistically significant was determine as * indicate p-value <0.05.

**Table S3. List of altered metabolites from pairwise O-PLS-DA analysis found in 2D culture and 3D MCS of CCA and cholangiocyte cell lines**

| Metabolite | LoA* | Molecular formula  [Adduct] | Retention time(min) | m/z observed | m/z  theoretical | ∆ppm | Mode | MMNK-1 | | KKU-213C | | KKU-213A | |
| --- | --- | --- | --- | --- | --- | --- | --- | --- | --- | --- | --- | --- | --- |
|  |  |  |  |  |  |  |  | **2D** | **3D** | **2D** | **3D** | **2D** | **3D** |
| Niacinamide | 2 | C_6_H_6_N_2_O  [M+H]^+^ | 2.03 | 123.055 | 122.048 | 2 | P | ↓ | ↑(*) | ↓ | ↑(*) | ↓ | ↑(*) |
| L-Methionine | 3 | C_5_H_11_NO_2_S  [M+H]^+^ | 1.32 | 150.058 | 149.051 | 2 | P | ↓ | ↑(*) | ↓ | ↑(*) | ↓ | ↑(*) |
| L-Tyrosine | 2 | C_9_H_11_NO_3_  [M+H]^+^ | 1.32 | 182.081 | 181.074 | 1 | P | ↓ | ↑(*) | ↓ | ↑(*) | ↓ | ↑(*) |
| Creatine | 2 | C4H9N3O2 [M+H]^+^ | 1.09 | 132.077 | 131.069 | 2 | P | - | - | ↓ | ↑ | ↑ | ↓ |
| L-Proline | 2 | C_5_H_9_NO_2_  [M+H]^+^ | 1.12 | 116.071 | 115.063 | 3 | P | ↓ | ↑(*) | ↓ | ↑(*) | - | - |
| L-Leucine | 2 | C_6_H_13_NO_2_ [M+H]^+^ | 1.32 | 132.102 | 131.095 | 1 | P | ↓ | ↑(*) | ↓ | ↑(*) | - | - |
| N,N,N-Trimethylethenaminium | 3 | C5H13NO  [M+H]^+^ | 1.00 | 104.107 | 103.099 | 1 | P | ↓ | ↑(*) | - | - | ↓ | ↑(*) |
| Cis-(homo)2aconitate | 3 | C_8_H_10_O_6_ [M+H]^+^ | 1.09 | 203.053 | 202.047 | 10 | P | - | - | - | - | ↓ | ↑(*) |
| Pyridoxine | 2 | C_8_H_11_NO_3_  [M+H] ^+^ | 1.33 | 170.081 | 169.074 | 1 | P | - | - | - | - | ↓ | ↑(*) |
| Phenylpyruvic acid | 3 | C_9_H_8_O_3_ [M+H] ^+^ | 1.33 | 165.054 | 164.047 | 4 | P | - | - | - | - | ↓ | ↑(*) |
| Phosphorylcholine | 3 | C_5_H_15_NO_4_P  [M+H] ^+^ | 1.07 | 184.073 | 184.074 | 2 | P | ↑(*) | ↓ | ↑(*) | ↓ | ↑(*) | ↓ |
| 2,5-Dichloro-4-oxohex-2-enedioic acid | 3 | C_6_H_4_Cl_2_O_5_  [M+H] ^+^ | 0.92 | 226.95 | 225.943 | 1 | P | - | - | ↑(*) | ↓ | ↑(*) | ↓ |
| Cysteinylglycine | 3 | C_5_H_10_N_2_O_3_S  [M+H] ^+^ | 1.33 | 179.05 | 178.041 | 3 | P | - | - | ↑(*) | ↓ | - | - |
| Phosphoric acid | 2 | H_3_O_4_P [M+H] ^+^ | 1.38 | 98.984 | 97.976 | 2 | P | - | - | - | - | ↑(*) | ↓ |
| 4-Methylene-L-glutamine | 3 | C_6_H_10_N_2_O_3_  [M+H] ^+^ | 1.07 | 159.08 | 158.069 | 9 | P | - | - | ↓ | ↑(*) | - | - |
| Indoline | 3 | C_8_H_9_N  [M+H] ^+^ | 4.82 | 120.081 | 119.073 | 2 | P | ↓ | ↑(*) | ↑ | ↓ | ↓ | ↑(*) |

*LoA: Level of Assignment; 1: Accurate mass matched to database, 2: Accurate mass matched to database and tandem MS spectrum matched to in silico fragmentation pattern, 3: Tandem MS spectrum matched to database or literature. P = ESI+ ionization mode, N= ESI- ionization mode, (↑) = High, (↓) = Low, (-) = Not found

**Table S3. List of altered metabolites from pairwise O-PLS-DA analysis found in 2D culture and 3D MCS of CCA and cholangiocyte cell lines** **(Cont.)**

| Metabolite | LoA* | | | Molecular  formula  [Adduct] | | Retention time(min) | m/z observed | m/z  theoretical | | ∆ppm | | Mode | MMNK-1 | | KKU-213C | | KKU-213A | |
| --- | --- | --- | --- | --- | --- | --- | --- | --- | --- | --- | --- | --- | --- | --- | --- | --- | --- | --- |
|  |  |  |  |  |  |  |  |  |  |  |  |  | **2D** | **3D** | **2D** | **3D** | **2D** | **3D** |
| Glycerophosphocholine | 2 | | | C_8_H_20_NO_6_P [M+H] ^+^ | | 1.06 | 258.11 | 257.102 | | 0 | | P | - | - | ↑ | ↓ | - | - |
| Decanoyl-CoA | 2 | | | C_32_H_48_N_11_O_15_PS_2_ [M+H] ^+^ | | 1.33 | 922.255 | 921.251 | | 4 | | P | - | - | ↑(*) | - | - | - |
| Betaine | 3 | | | C_5_H_11_NO_2_  [M+H] ^+^ | | 1.08 | 118.086 | 117.079 | | 2 | | P | ↑ | ↓ | - | - | ↓ | ↑(*) |
| L-phenylalnine | 2 | | | C_9_H_11_NO_2_  [M+H] ^+^ | | 4.81 | 166.086 | 165.078 | | 2 | | P | ↓ | ↑(*) | - | - | ↓ | ↑(*) |
| L-Arginine | 3 | | | C_6_H_14_N_4_O_2_  [M+H] ^+^ | | 0.97 | 175.119 | 174.112 | | 1 | | P | ↓ | ↑(*) | - | - | ↓ | ↑(*) |
| Indoleacrylic acid | 3 | | | C_11_H_9_NO_2_  [M+H] ^+^ | | 5.68 | 188.071 | 187.063 | | 2 | | P | ↓ | ↑(*) | - | - | ↓ | ↑ |
| L-Acetylcarnitine | 2 | | | C_9_H_17_NO_4_  [M+H] ^+^ | | 1.25 | 204.123 | 203.116 | | 0 | | P | ↓ | ↑(*) | ↑(*) | ↓ | - | - |
| L-Tryptophan | 2 | | | C_11_H_12_N_2_O_2_  [M+H] ^+^ | | 5.68 | 205.097 | 204.089 | | 1 | | P | ↓ | ↑(*) | - | - | - | - |
| Glycerol | 3 | | | C3H8O3 [M+H] ^+^ | | 1.10 | 93.0546 | 92.047 | | 0 | | P | ↑ | ↓ | - | - | - | - |
| 6-Hydroxynicotinic acid | 3 | | | C_6_H_5_NO_3_ [M+H] ^+^ | | 21.02 | 140.033 | 139.027 | | 9 | | P | ↑ | ↓ | - | - | - | - |
| 2,4-Dichloro-3-oxoadipate | 3 | | | C_6_H_4_Cl_2_O_5_-^2^ [M+H] ^+^ | | 0.92 | 226.951 | 225.943 | | 1 | | P | ↑(*) | ↓ | - | - | - | - |
| Oleamide | | 3 | C_18_H_35_NO  [M+H] ^+^ | | 17.76 | | 282.279 | | 281.272 | | 0 | P | ↑(*) | ↓ | - | - | - | - |
| L-Glutamine | | 2 | C_5_H_10_N_2_O_3_  [M+H] ^+^/ [M-H] ^-^ | | 1.09/1.08 | | 147.076/  145.062 | | 146.069 | | 3/1 | P/N | ↓ | P↑(*) | ↓ | ↑(*) | ↓ | ↑(*) |
| N-Acryloylglycine | | 3 | C5H7NO3 [M+H] ^+^/ [M-H] ^-^ | | 1.31/1.34 | | 130.050/  128.035 | | 129.043 | | 1/2 | P/N | ↓ | N↑(*) | ↓ | ↑(*) | ↓ | ↑ |
| Pyroglutamic acid | | 3 | C_5_H_7_NO_3_ [M+H] ^+^/ [M-H] ^-^ | | 2.02/1.92 | | 130.050/  128.036 | | 129.042 | | 1/5 | P/N | - | - | ↓ | N↑(*) | ↓ | P↑(*) |
| Glutathione | | 2 | C_10_H_17_N_3_O_6_S  [M+H] ^+^/ [M-H]^-^ | | 2.00/1.88 | | 308.091/  306.076 | | 307.084 | | 0/1 | P/N | ↑(*) | ↓ | ↑(*) | ↓ | P ↑(*) | ↓ |
| Adenosine 5'-monophosphate (AMP) | | 3 | C_12_H_9_N_7_O_6_  [M+H] ^+^/ [M-H]^-^ | | 1.34/1.36 | | 348.07/  346.057 | | 347.061 | | 1/3 | P/N | - | - | P↑(*) | ↓ | - | - |
| L-Valine | | 3 | C_5_H_11_NO_2_  [M+H] ^+^/ [M-H]^-^ | | 1.24/1.24 | | 118.086/116.072 | | 117.079 | | 2/3 | P/N | ↓ | ↑(*) | ↓ | P ↑(*) | ↓ | ↑(*) |
| Citrulline | | 3 | C_6_H_13_N_3_O_3_ [M+H] ^+^/ [M-H]^-^ | | 1.07/1.06 | | 174.095/174.089 | | 175.096 | | 3/3 | P/N | - | - | ↓ | ↑(*) | - | - |

*LoA: Level of Assignment; 1: Accurate mass matched to database, 2: Accurate mass matched to database and tandem MS spectrum matched to in silico fragmentation pattern, 3: Tandem MS spectrum matched to database or literature. P = ESI+ ionization mode, N= ESI- ionization mode, (↑) = High, (↓) = Low, (-) = Not found

**Table S3. List of altered metabolites from pairwise O-PLS-DA analysis found in 2D culture and 3D MCS of CCA and cholangiocyte cell lines (Cont.)**

| Metabolite | LoA* | | Molecular  formula  [Adduct] | Retention time(min) | | m/z observed | | m/z  theoretical | | ∆ppm | Mode | MMNK-1 | | KKU-213C | | KKU-213A | |
| --- | --- | --- | --- | --- | --- | --- | --- | --- | --- | --- | --- | --- | --- | --- | --- | --- | --- |
|  |  |  |  |  |  |  |  |  |  |  |  | **2D** | **3D** | **2D** | **3D** | **2D** | **3D** |
| Hypoxanthine | 3 | | C_5_H_4_N_4_O [M+H] ^+^/ [M-H]^-^ | 1.33/1.34 | | 137.046/135.031 | | 136.039 | | 2/2 | P/N | ↓ | P↑(*) | ↓ | N↑(*) | - | - |
| L-Lactic acid | 3 | | C_3_H_6_O_3_ [M-H]^-^ | 1.79 | | 89.024 | | 90.031 | | 3 | N | ↓ | ↑(*) | ↓ | ↑(*) | ↓ | ↑(*) |
| L-Threonine | 2 | | C_4_H_9_NO_3_ [M-H]^-^ | 1.09 | | 118.051 | | 119.058 | | 0 | N | - | - | ↓ | ↑(*) | ↓ | ↑(*) |
| L-Glyceraldehyde | 3 | C_3_H_6_O_3_  [M-H]^-^ | | | 1.38 | 89.0241 | 90.032 | | 3 | | N | ↓ | ↑(*) | ↓ | ↑(*) | - | - |
| 3-Phosphoglyceric acid | 2 | C_3_H_7_O_7_P  [M-H]^-^ | | | 1.48 | 184.986 | 185.993 | | 2 | | N | ↓ | ↑(*) | ↓ | ↑(*) | - | - |
| 3-Hydroxypropanoic acid | 3 | C_3_H_6_O_3_  [M-H]^-^ | | | 1.56 | 89.0241 | 90.032 | | 3 | | N | - | - | ↓ | ↑(*) | - | - |
| Fructose 1,6-bisphosphate | 2 | C_6_H_14_O_12_P_2_ [M-H]^-^ | | | 1.48 | 338.989 | 339.996 | | 1 | | N | ↓ | ↑(*) | - | - | ↓ | ↑(*) |
| Ureidoisobutyric acid | 3 | C_5_H_10_N_2_O_3_ [M-H]^-^ | | | 0.98 | 145.062 | 146.069 | | 1 | | N | - | - | - | - | ↓ | ↑(*) |
| 6-Phosphogluconic acid | 2 | C_6_H_13_O_10_P  [M-H]^-^ | | | 1.46 | 275.018 | 276.025 | | 2 | | N | - | - | - | - | - | ↑(*) |
| Adenosine 5'-diphosphate (ADP) | 3 | C_10_H_15_N_5_O_10_P_2_  [M-H]^-^ | | | 1.73 | 426.023 | 427.029 | | 2 | | N | ↑(*) | ↓ | ↑(*) | ↓ | ↑(*) | - |
| Uridine 5'-diphosphate (UDP) | 3 | C_10_H_10_N_6_O_8_P_2_ [M-H]^-^ | | | 1.6 | 402.996 | 404.002 | | 3 | | N | - | - | - | - | ↑(*) | ↓ |
| L-Glutamic acid | 2 | C_5_H_9_NO_4_  [M-H]^-^ | | | 1.06 | 146.046 | 147.053 | | 2 | | N | ↑(*) | ↓ | - | - | ↑(*) | ↓ |
| L-Aspartic acid | 3 | | C_4_H_7_NO_4_  [M-H]^-^ | 1.06 | | 132.030 | 133.037 | | 2 | | N | - | - | - | - | ↑(*) | ↓ |
| Phosphodimethylethanolamine | 3 | | C_4_H_12_NO_4_P [M-H]^-^ | 1.06 | | 168.043 | 169.050 | | 1 | | N | - | - | - | - | ↑(*) | ↓ |
| Uridylic acid (UMP) | 3 | | C_9_H_13_N_2_O_9_P [M-H]^-^ | 1.40 | | 323.029 | 324.035 | | 1 | | N | ↑(*) | ↓ | - | - | - | - |
| L-Ornithine | 3 | | C5H12N2O2 [M-H]^-^ | 1.08 | | 131.083 | 132.089 | | 3 | | N | - | - | ↓ | ↑(*) | - | - |

*LoA: Level of Assignment; 1: Accurate mass matched to database, 2: Accurate mass matched to database and tandem MS spectrum matched to in silico fragmentation pattern, 3: Tandem MS spectrum matched to database or literature. P = ESI+ ionization mode, N= ESI- ionization mode, (↑) = High, (↓) = Low, (-) = Not found

**Table S3. List of altered metabolites from pairwise O-PLS-DA analysis found in 2D culture and 3D MCS of CCA and cholangiocyte cell lines (Cont.)**

| Metabolite | LoA* | Molecular  formula  [Adduct] | Retention time(min) | m/z observed | m/z  theoretical | ∆ppm | Mode | MMNK-1 | | KKU-213C | | KKU-213A | |
| --- | --- | --- | --- | --- | --- | --- | --- | --- | --- | --- | --- | --- | --- |
|  |  |  |  |  |  |  |  | **2D** | **3D** | **2D** | **3D** | **2D** | **3D** |
| Xanthine | 2. | C_5_H_4_N_4_O_2_  [M-H]^-^ | 1.34 | 151.026 | 152.033 | 1 | N | - | - | ↓ | ↑(*) | - | - |
| Inosine | 2 | C_10_H_12_N_4_O_5_ [M-H]^-^ | 1.36 | 267.074 | 268.08 | 2 | N | - | - | ↓ | ↑(*) | - | - |
| Taurine | 2 | C_2_H_7_NO_3_S [M-H]^-^ | 1.05 | 124.008 | 125.014 | 5 | N | - | - | ↓ | ↑ | - | - |
| UDP-N-Acetylglucosamine | 3 | C_17_H_27_N_3_O_17_P_2_  [M-H]^-^ | 1.51 | 606.075 | 607.081 | 1 | N | ↓ | ↑(*) | ↑ | ↓ | ↑(*) | ↓ |
| Citric acid | 2 | C_6_H_8_O_7_  [M-H]^-^ | 1.38 | 191.02 | 192.027 | 1 | N | ↓ | ↑(*) | ↑(*) | ↓ | - | - |
| Methylmalonic acid | 2 | C_4_H_6_O_4_ [M-H]^-^ | 1.45 | 117.019 | 118.027 | 3 | N | - | - | ↑(*) | ↓ | - | - |
| Fumaric acid | 3 | C_4_H_2_O_4_ [M-H]^-^ | 0.91 | 112.986 | 116.011 | 3 | N | - | - | - | - | ↑(*) | ↓ |
| N-Acetyl-L-aspartic acid | 2 | C_5_H_7_NO_3_  [M-H]^-^ | 1.34 | 128.035 | 129.042 | 2 | N | ↑(*) | ↓ | - | - | - | - |

*LoA: Level of Assignment; 1: Accurate mass matched to database, 2: Accurate mass matched to database and tandem MS spectrum matched to in silico fragmentation pattern, 3: Tandem MS spectrum matched to database or literature. P = ESI+ ionization mode, N= ESI- ionization mode, (↑) = High, (↓) = Low, (-) = Not found


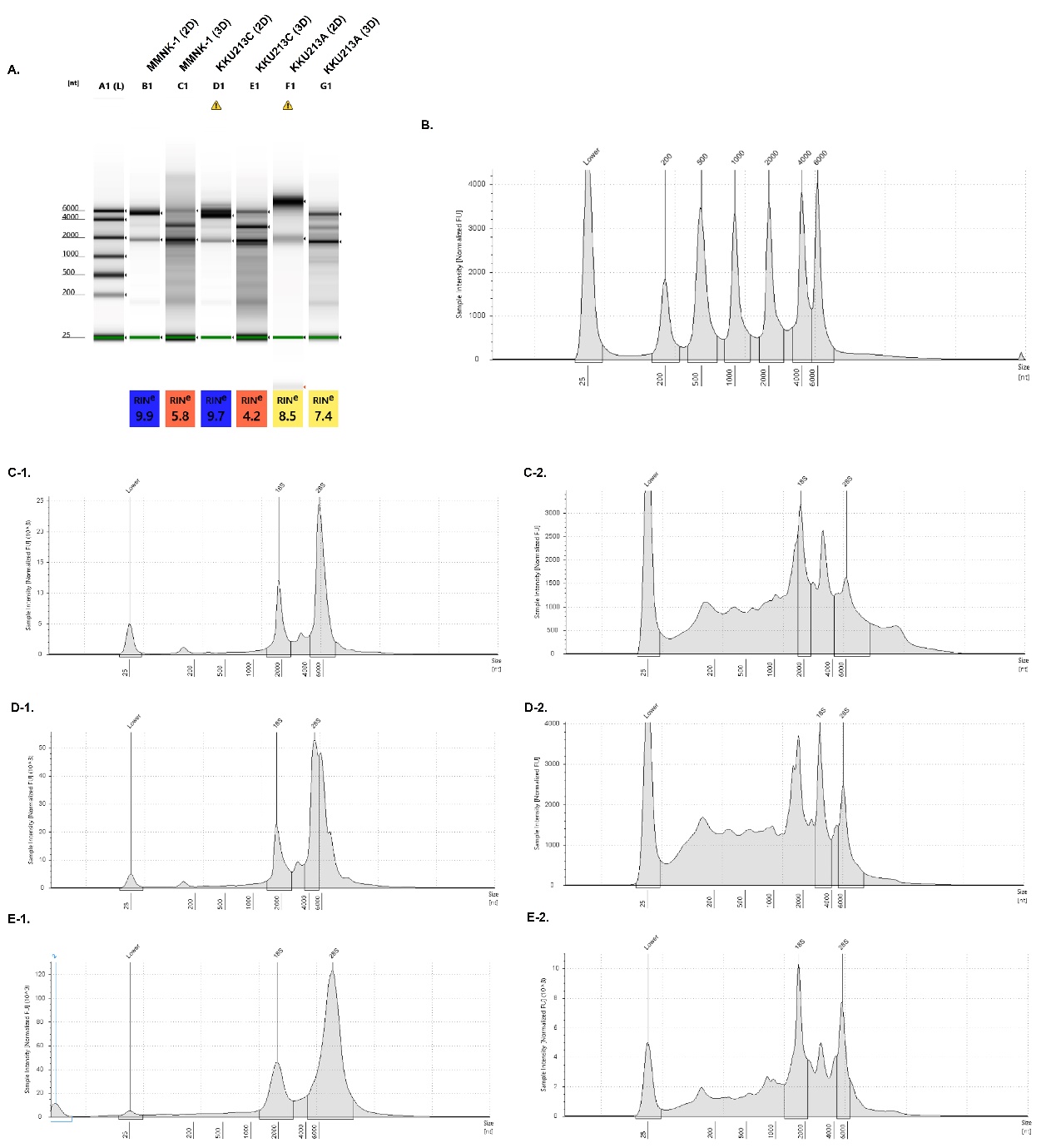


**Figure S13.** RNA integrity analysis showing electropherogram and RNA integrity number (RIN) of all samples (A), markers (B), the normalized intensity of 2D and 3D MMNK-1 (C-1, C-2), the normalized intensity of 2D and 3D KKU-213C (D-1, D-2), and the normalized intensity 2D and 3D KKU-213A (E-1, E-2). The results showed the RNA integrity of 3D MCSs were reduced, presenting with the higher amount of shorter fragments of RNA and the reduced ratio between 28S and 18S RNAs.


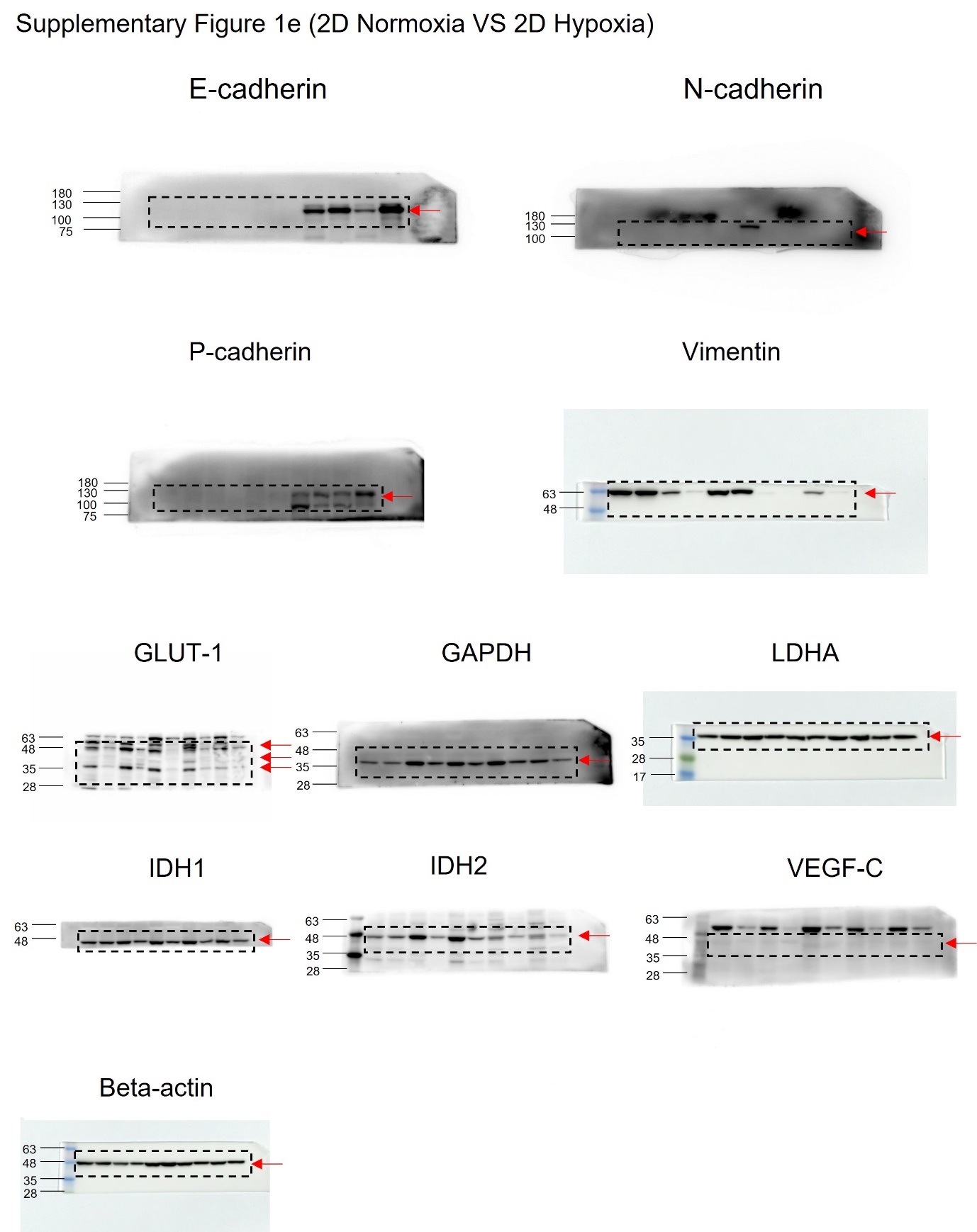


**Figure S14.** Western blot analysis of cell adhesion molecules (E-cadherin, N-cadherin, and P-cadherin), mesenchymal marker and enzymes involved in the glycolytic pathway (GLUT-1, GAPDH, and LDHA), enzymes related to TCA cycle (IDH1 and IDH2) and angiogenic activity (VEGF-C) expressexpress on 2D nornoxia VS 2D hypoxia of CCA and cholangiocyte cell lines.


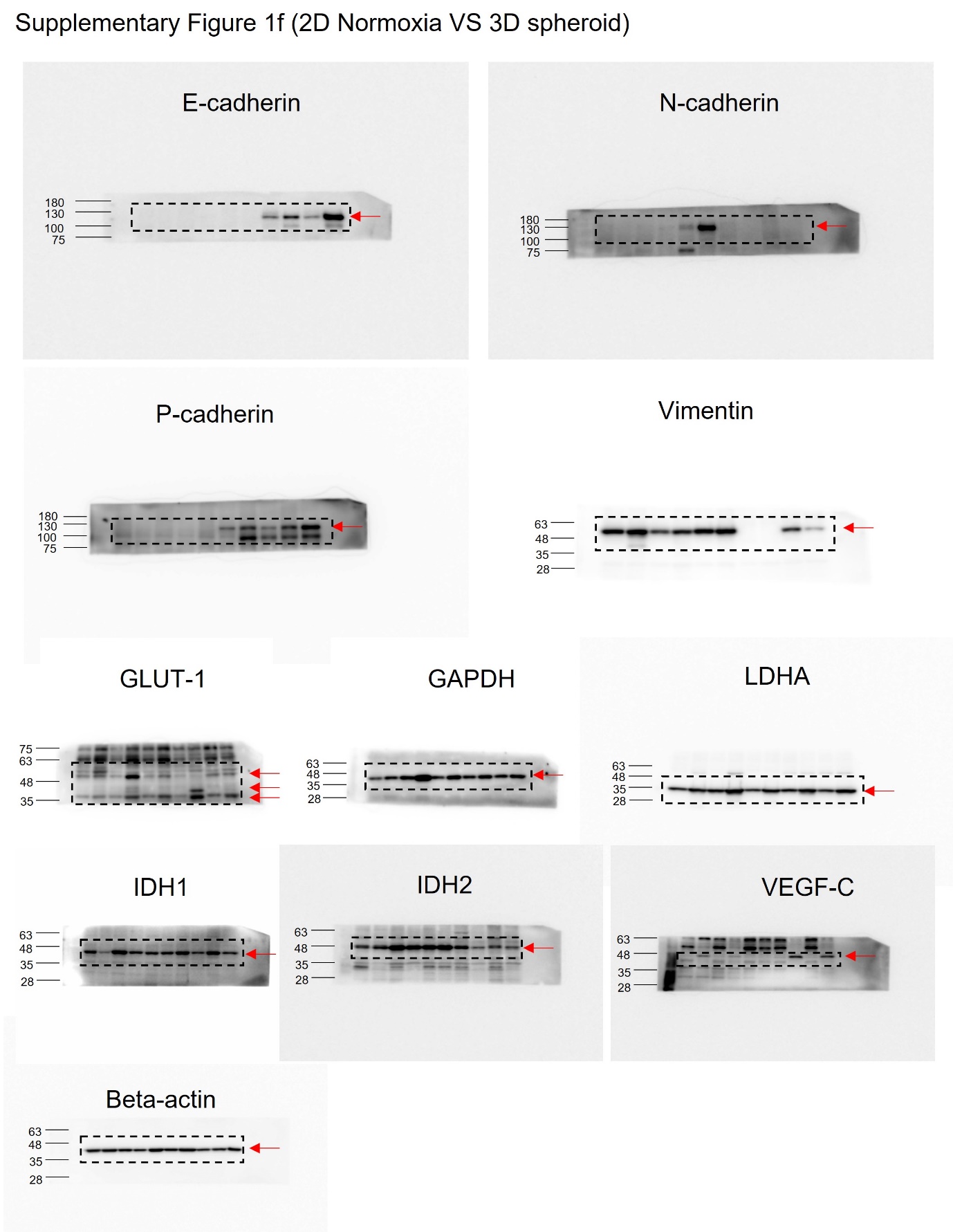


**Figure S15.** Western blot analysis of cell adhesion molecules (E-cadherin, N-cadherin, and P-cadherin), mesenchymal marker and enzymes involved in the glycolytic pathway (GLUT-1, GAPDH, and LDHA), enzymes related to TCA cycle (IDH1 and IDH2) and angiogenic activity (VEGF-C) expressexpress on 2D nornoxia VS 3D spheroid of CCA and cholangiocyte cell lines.


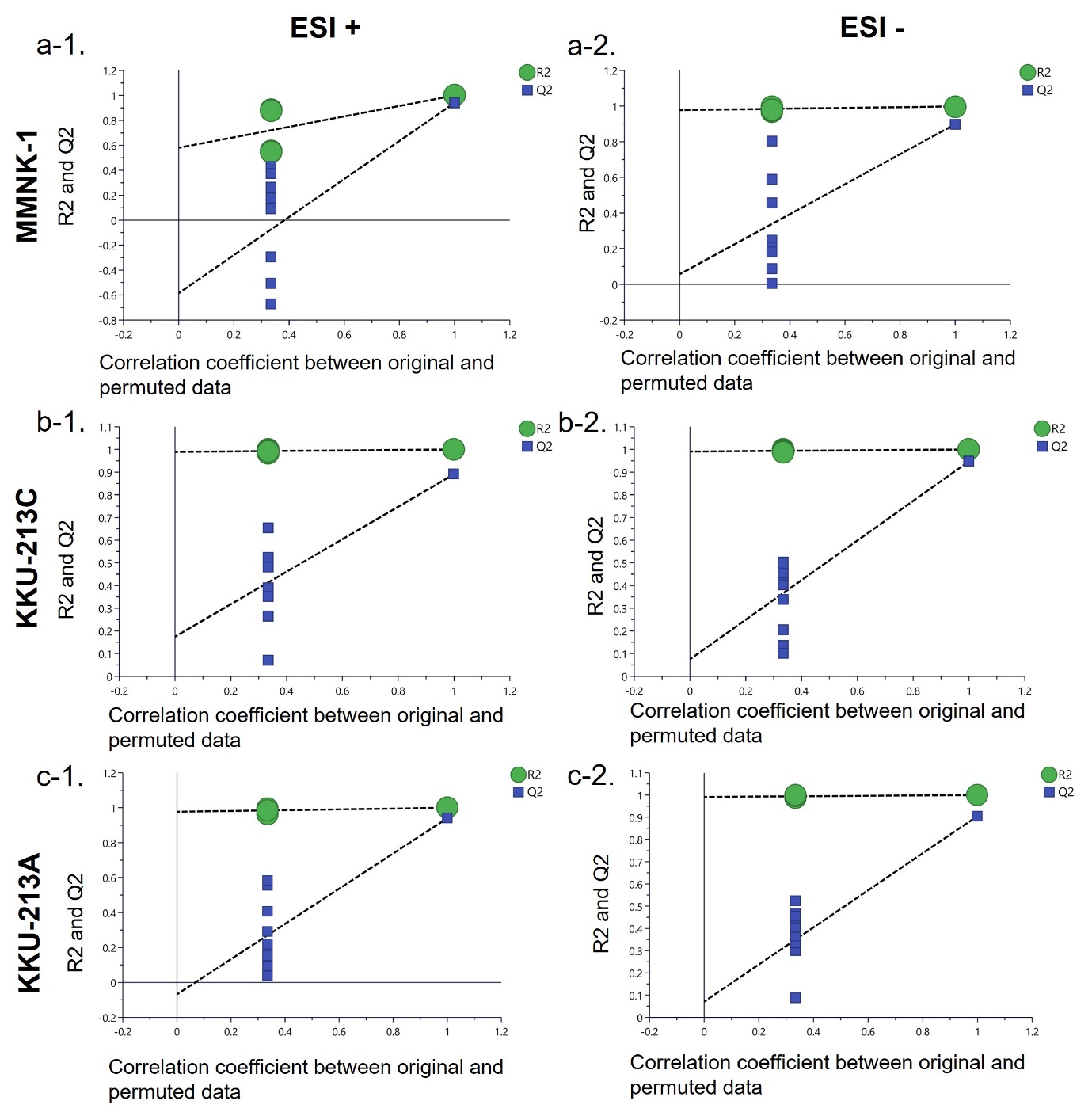


**Figure S16.** Permutation test of O-PLS-DA models between 2D and 3D MMNK-1 (a), KKU-213C (b), and KKU-213A (c) in 1) positive and 2) negative ESI modes.


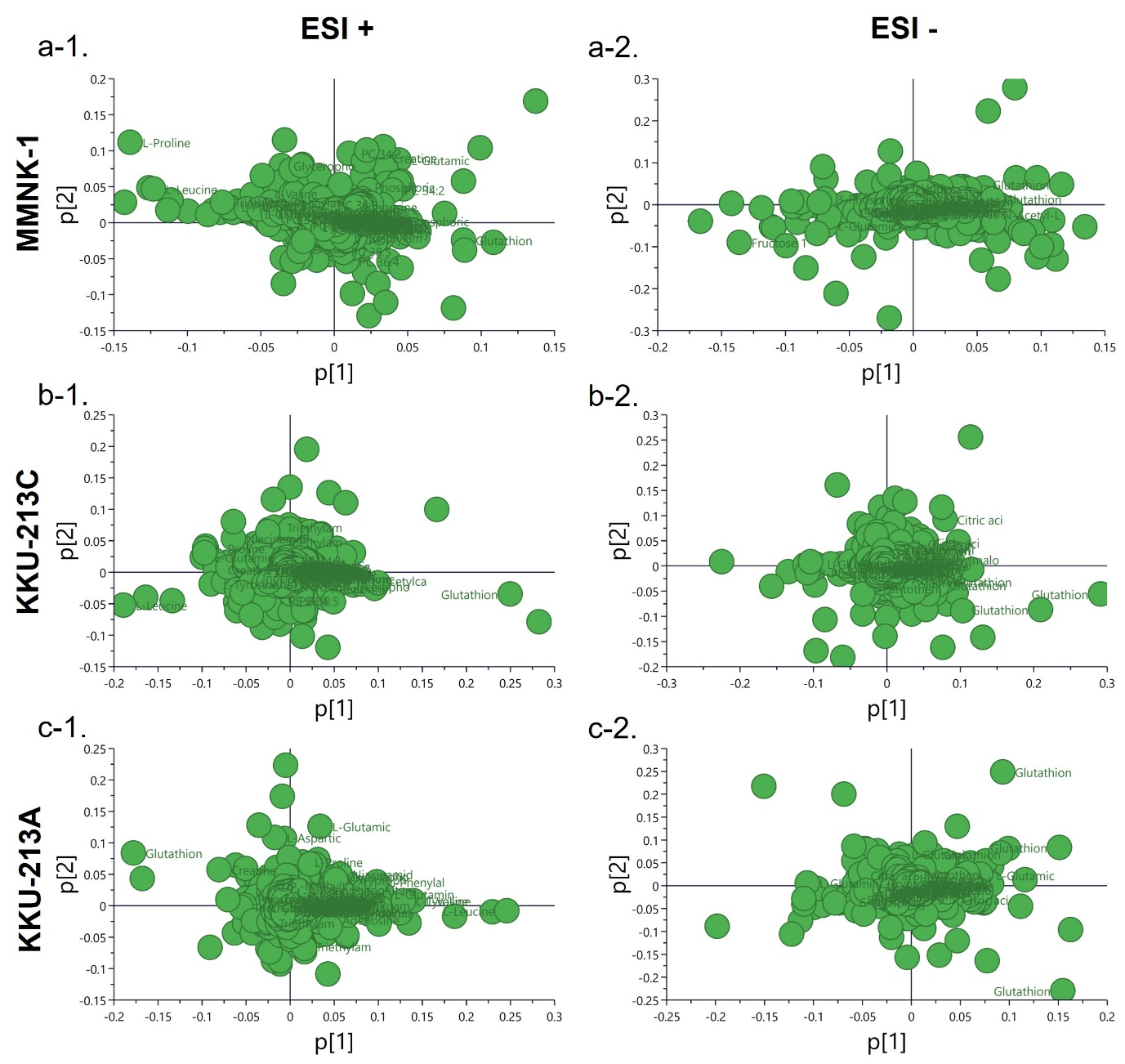


**Figure S17.** PCA loading plots of 2D and 3D metabolic profiles of MMNK-1 (a), KKU-213C (b) and KKU-213A (c) in 1) positive and 2) negative ESI modes.
